# Exosomal miR-146a-5p derived from human umbilical cord mesenchymal stem cells can alleviate antiphospholipid antibody-induced trophoblast injury and placental dysfunction by regulating the TRAF6/NF-κB axis

**DOI:** 10.1186/s12951-023-02179-5

**Published:** 2023-11-13

**Authors:** Qingfeng Lv, Yuan Wang, Wei Tian, Yuqiu Liu, Mengqi Gu, Xiaotong Jiang, Yanjun Cai, Ruiheng Huo, Yuchen Li, Lei Li, Xietong Wang

**Affiliations:** 1 Department of Obstetrics and Gynecology, Shandong Provincial Hospital Affiliated to Shandong First Medical University, Jinan, 250021 Shandong China; 2 The Laboratory of Medical Science and Technology Innovation Center (Institute of Translational Medicine), Shandong First Medical University (Shandong Academy of Medical Sciences) of China, Jinan, 250117 Shandong China; 3 The Key Laboratory of Birth Regulation and Control Technology of National Health Commission of China, Shandong Provincial Maternal and Child Health Care Hospital, Jinan, 250014 Shandong China

**Keywords:** Obstetric antiphospholipid syndrome, Antiphospholipid antibodies, Umbilical cord mesenchymal stem cells, Exosomes, miR-146a-5p

## Abstract

Exosomes originating from human umbilical cord mesenchymal stem cells (hucMSC-exos) have become a novel strategy for treating various diseases owing to their ability to regulate intercellular signal communication. However, the potential of hucMSC-exos to improve placental injury in obstetric antiphospholipid syndrome and its underlying mechanism remain unclear. Our objective was to explore the potential application of hucMSC-exos in the treatment of obstetric antiphospholipid syndrome and elucidate its underlying mechanism. In our study, hucMSC-exos ameliorated the functional impairment of trophoblasts caused by antiphospholipid antibodies in vitro and attenuated placental dysfunction in mice with obstetric antiphospholipid syndrome by delivering miR-146a-5p. Exosomal miR-146a-5p suppressed the expression of tumor necrosis factor receptor-associated factor 6 (TRAF6) and inhibited the activation of NF-κB signaling, leading to the down-regulation of IL-1β and IL-18 to rescue inflammation and modulation of Cleaved-CASP3, BAX, and BCL2 to inhibit apoptosis in HTR8/SVneo cells and mice placenta. This study identified the potential molecular basis of how hucMSC-exos improved antiphospholipid antibody-induced placental injury and highlighted the functional importance of the miR-146a-5p/TRAF6 axis in the progression of obstetric antiphospholipid syndrome. More importantly, this study provided a fresh outlook on the promising use of hucMSC-exos as a novel and effective treatment approach in obstetric antiphospholipid syndrome.

**Graphical Abstract:**

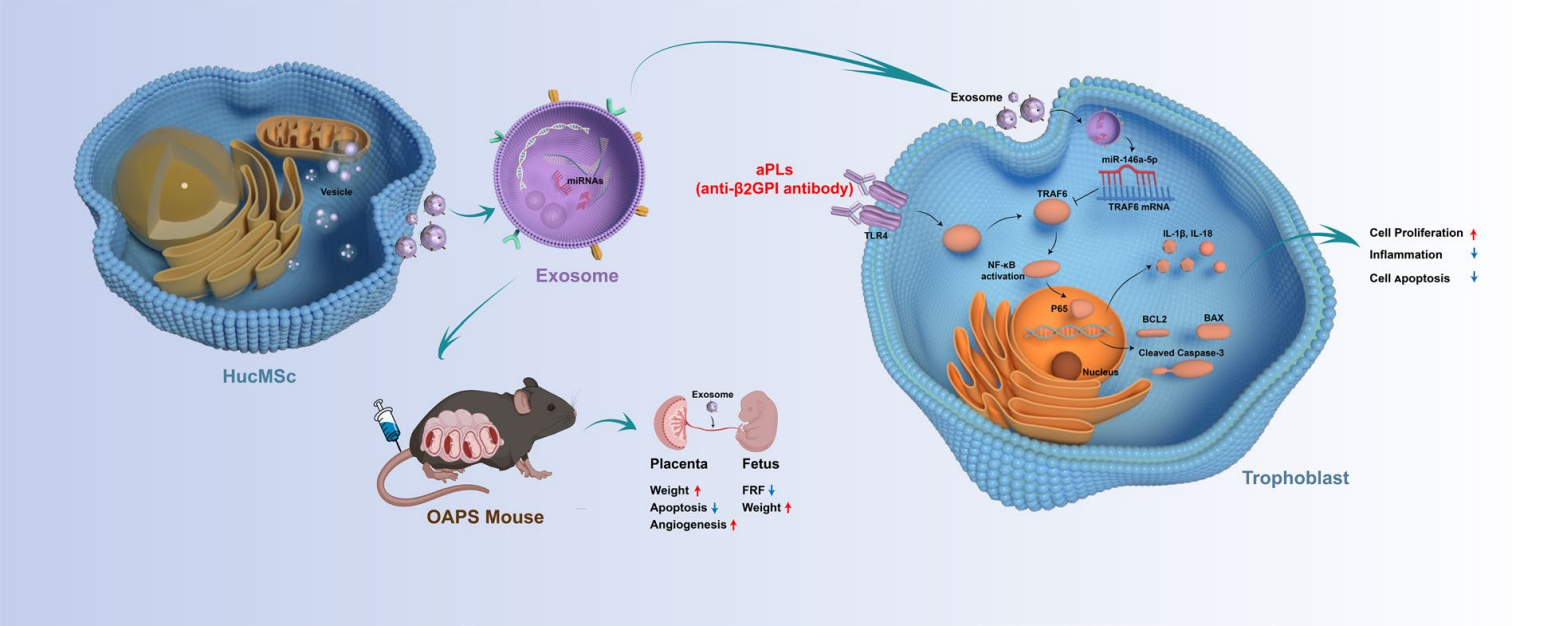

**Supplementary Information:**

The online version contains supplementary material available at 10.1186/s12951-023-02179-5.

## Background

Antiphospholipid syndrome (APS) is an autoimmune disorder that occurs when circulating antiphospholipid antibodies (aPLs) lead to thrombogenesis and/or pathological pregnancy [1, 2]. APS is referred to as obstetric antiphospholipid syndrome (OAPS) when pathological pregnancy is the main manifestation, such as recurrent abortion, stillbirth, preterm delivery, pre-eclampsia, and fetal growth restriction [3]. Besides a history of at a minimum one clinical symptom, continuous and stable existence of one or more autoantibodies is also necessary: anti-cardiolipin antibodies, anti-β2-glycoprotein I (β2GPI) antibodies, or a lupus anticoagulant. The presence of β2GPI on the surfaces of trophoblasts and decidual endothelial cells is constant, making the placenta susceptible to harmful β2GPI binding, which interferes with the proliferation and invasion of trophoblasts and destroys interaction between trophoblasts and endothelial cells, resulting in spiral artery remodeling fails and placental dysfunction, increasing the risk of adverse maternal or fetal outcomes [4–7]. Although the pathogenesis of OAPS is not fully understood, many studies have shown that placental inflammation [8, 9], utero-placental circulation deficiency [10, 11] and placental apoptosis [12] are the hallmark characteristic of poor obstetric outcomes. Previous prospective observational studies reported that patients with APS experience 10–12% of fetal death rates and 9–10% of pre-eclampsia with severe features [13, 14].

The present recommended therapy for OAPS primarily involves the use of aspirin in conjunction with low-molecular-weight heparin, with hormone and hydroxychloroquine being incorporated as needed. However, irreversible placental damage still occurs in some patients with refractory APS, leading to adverse pregnancy outcomes and serious obstetric complications [15–17]. Moreover, the current treatment for OAPS focuses on anticoagulation and ignores its underlying pathophysiological mechanisms; therefore, new treatment approaches are needed to reverse placental damage, improve pregnancy outcomes, and prevent pregnancy complications.

Exosomes—disk-like nanovesicles with a lipid bilayer and diameter of 30–150 nm involved in diverse biological processes—are important mediators of intercellular interaction [18]. Since exosomes can naturally carry intercellular molecules, they have become therapeutic carriers and can be used to treat different types of diseases [19]. Researchers have explored the medical value of exosomes derived from mesenchymal stem cells due to their effectiveness in regenerative medicine, lack of immune response, minimal ethical concerns and low rates of embolism and carcinogenesis [20–24]. Numerous studies have demonstrated that exosomes originating from human umbilical cord mesenchymal stem cells (hucMSC-exos) exert different biological effects in diverse disorders, such as anti-inflammatory [25–27], anti-apoptosis [28–30], tissue repair [31, 32], angiogenesis [33, 34] and immune regulation [35, 36]. Recent research has reported that hucMSC-exos can inhibit the occurrence and progression of pre-eclampsia by promoting the proliferation, migration, and invasion abilities of trophoblasts, inhibiting apoptosis and improving angiogenesis [37–39]. However, scientists are yet to elucidate whether hucMSC-exos can restore aPL-mediated trophoblast injury and placental impairment in OAPS.

MiRNA is a small non-coding RNA with a length of 18–22 nt, which has recently been identified as a key component in regulating various pathological processes [40]. Previous study has shown that miRNA can be secreted and delivered to specific effector cells for functional regulation, mainly by regulating post-transcriptional gene expression [41]. Numerous investigations have highlighted the essential role of certain miRNAs found abundantly in hucMSC-exos in facilitating healing processes across various illness. Exosomal miR-146a-5p and miR-221-3p have been shown to ameliorate hypertrophy of the ligamentum flavum [42]. Additionally, miR-377-3p derived from hucMSCs-exos has been verified to alleviate both in vivo and in vitro acute lung injury [43]. These small non-coding RNAs can inhibit the translation of transcripts by suppressing the expression of target genes, thereby regulating important biological processes, including pathological pregnancy. Furthermore, miR-101 encapsulated in exosomes inhibited the activation of the NF-κB/CXCL11 pathway through suppression the BRD4 expression, consequently enhancing the proliferation and migration of trophoblast cells in pre-eclampsia [38]. HucMSC-exos-derived miR-18b [39] and miR-133b [44] promoted trophoblasts proliferation and migration by downregulating TIM3 and SGK1, respectively. Furthermore, it has been discovered that hucMSC-exos can reduce the damage caused to endometrial epithelial cells by hypoxia through the regulation of the miR-663a/CDKN2A axis [45]. Nonetheless, it remains unclear whether the specific miRNAs derived from hucMSC-exos could be served as an innovative therapeutic approach for OAPS.

The objective of this research was to identify the potential application of hucMSC-exos in the treatment of OAPS, and to further validate the potential mechanisms of specific miRNAs derived from hucMSC-exos in OAPS.

## Results

### Isolation and characterization of hucMSCs and hucMSC-exos

**Fig. 1:**
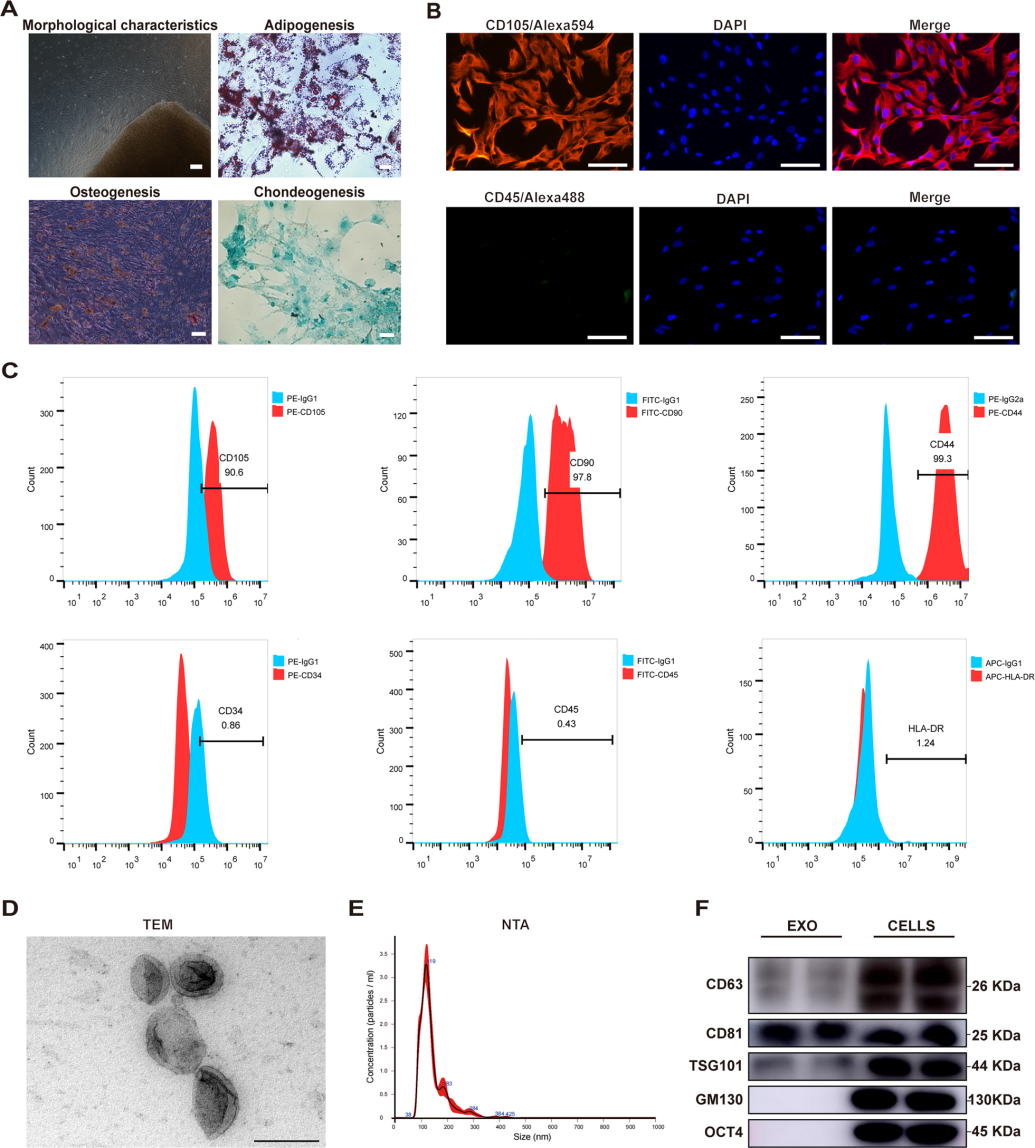
Isolation and characterization of human umbilical cord mesenchymal stem cells (hucMSCs) and hucMSC-exos. **A** The morphology of hucMSCs isolated and cultured by tissue block attachment at the third passage (100x), the Oil Red O staining of adiopogenic identification (400x), the Alizarin Red staining of osteogenic identification (100x) and the Alcian blue staining of chondrogenic differentiation (200x). **B** Immunofluorescence (IF) staining of CD105 and CD45 of hucMSCs. Scale bars, 50 μm. **C** Positive expression of CD105, CD90, and CD44 and negative expression of CD34, CD44, and HLA-DR were detected by flow cytometry. The blue peak represented the isotype control antibody and the red one represented the MSC-associated markers. **D** Representative transmission electron microscopy (TEM) image of hucMSC-exos. Scale bar, 200 nm. **E** The particle size and concentration of hucMSC-exos measured by nanoparticle tracking analysis (NTA). **F** Representative western blot images of exosomal-associated (CD63, CD81, TSG101), Golgi-associated (GM130) and MSC-associated (OCT4) markers in hucMSC-exos and hucMSCs

First, hucMSCs were successfully isolated and confirmed using adipogenic, osteogenic, and chondrogenic differentiation assays (Fig. 1A). Immunofluorescence staining showed that CD105 was highly expressed on the surface, and CD45 was undetected (Fig. 1B). Additionally, CD105, CD90 and CD44 showed high positive expression, CD34, CD45, and HLA-DR were negatively expressed, as detected by flow cytometry (Fig. 1C). Subsequently, hucMSC-exos were extracted using a step-by-step ultracentrifugation program and verified using the guiding principles of the ISEV [46]. Transmission electron microscopy (TEM) and nanoparticle tracking analysis (NTA) showed that hucMSC-exos were 30–150 nm in diameter with 1.84e + 11 ± 1.9e + 10 particles/mL concentration and a classical saucer shape double-layer membrane structure (Fig. 1D and E). The features of hucMSC-exos were further verified using western blot assay (Fig. 1F), and the specific exosomal biomarkers CD63, CD81, and TSG101 were detected compared with Golgi marker (GM130) and MSC-associated protein (OCT4).

### HucMSC-exos dramatically ameliorate the functional impairment of HTR8/SVneo cells caused by aPL in vitro

**Fig. 2:**
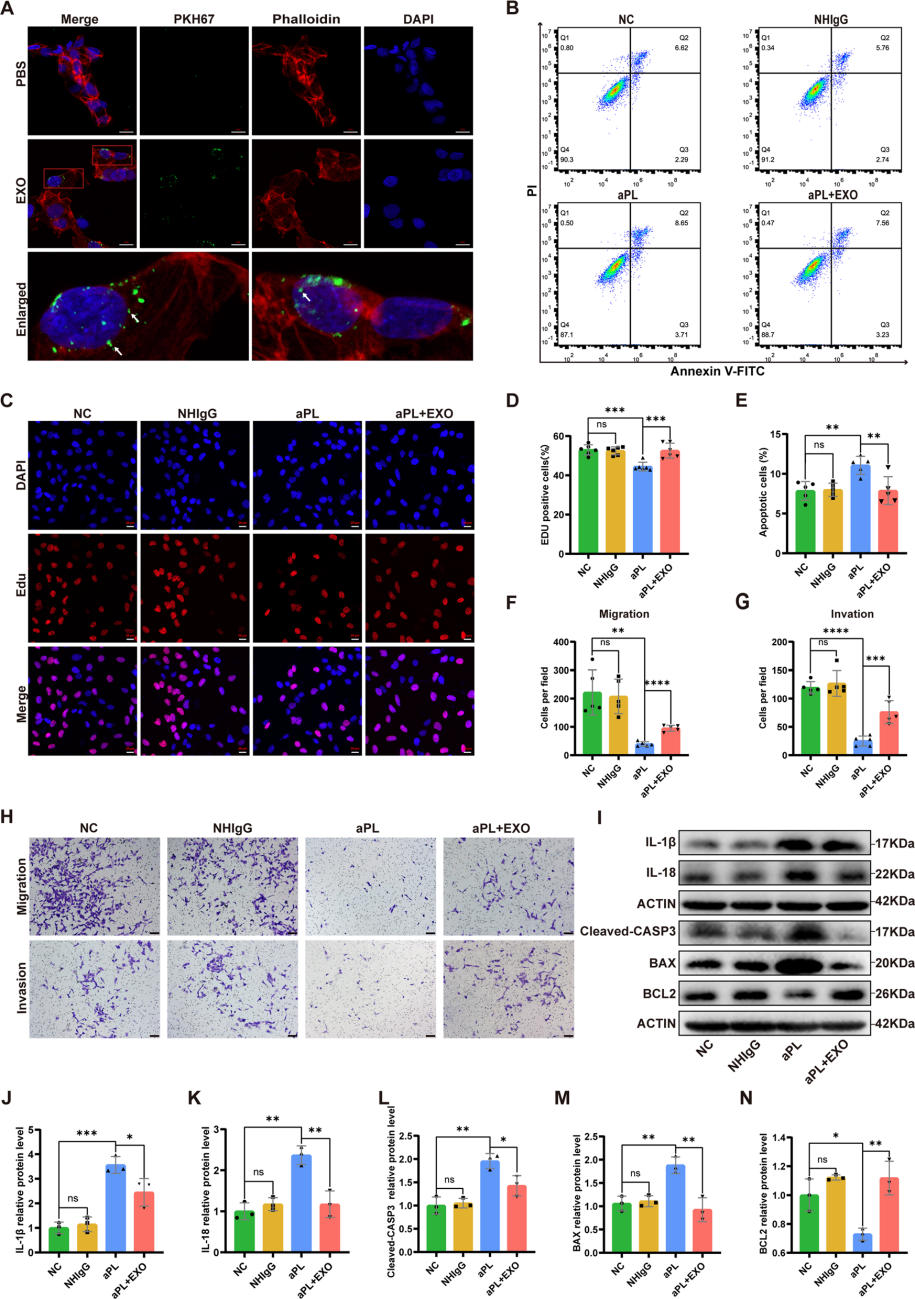
HucMSC-exos ameliorated the functional impairment of HTR8/SVneo cells caused by antiphospholipid antibody (aPL) in vitro. **A** Representative images of internalization of hucMSC-exos in HTR8/SVneo cells in vitro. Exosomes were labeled by PKH67, the cytoskeleton was stained with phalloidin-iFluor 594 and nuclei were counterstained with DAPI. Scale bars, 20 μm. **B-N** HTR8/SVneo cells were treated with NHIgG or aPL (200 μg/mL) for 24 h, and then hucMSC-exos were added into the medium (100 μg/mL) for 24 h. **B** and **E** The cell apoptotic rate was determined by flow cytometry analysis with annexin-V/PI staining (n = 5). **C** and **D** The proliferation ability of HTR8/SVneo cells in different treatment groups was detected by EdU assay (n = 6). Scale bars, 20 μm. **F-H** Transwell experiments were performed to measure the abilities of migration and invasion of HTR8/SVneo cells, hucMSC-exos improved the abilities of migration and invasion of HTR8/SVneo cells pretreated with aPL (n = 5). Scale bars, 100 μm. **I-N** Western blot analyzed the relative levels of apoptosis-related (Cleavd-CASP3, BAX, BCL2) and inflammation-associated (IL-1β, IL-18) proteins in HTR8/SVneo cells under different treatment conditions, quantified by signal intensity normalized to ACTIN (n = 3). *p < 0.05, **p < 0.01, ***p < 0.001, ****p < 0.0001

To assess the effect of exosomes on aPL-induced cell injury, hucMSC-exos were co-cultured with aPL-pretreated HTR8/SVneo cells, and diverse cell function experiments were performed. Additionally, IgG was purified from normal healthy controls and patients with APS, termed NHIgG and aPL, respectively. Exosomes labeled with PKH67 could been taken up by HTR8/SVneo cells (Fig. 2A). Compared with the negative control (NC) and NHIgG groups, cells in the aPL group showed a decreased cell proliferation rate, which was reversed by hucMSC-exos, as determined using EdU assay (Fig. 2C and D). Consistent with the EdU assay, flow cytometry showed that aPL induced apoptosis in HTR8/SVneo cells, which was markedly inhibited by hucMSC-exos (Fig. 2B and E). Furthermore, cell migration and invasion experiments revealed that aPL could impair the migration and invasion abilities of HTR8/SVneo cells, which could be ameliorated by hucMSC-exos (Fig. 2F-H). Western blot analysis revealed that exosome intervention markedly reduced the level of apoptosis-related proteins (Cleaved-CASP3 and BAX) and upregulated the level of the anti-apoptotic protein BCL2 (Figs. 2I-N). Lastly, hucMSC-exos significantly reduced inflammatory factors IL-1β and IL-18. These data suggest that hucMSC-exos could protect HTR8/SVneo cells from functional injury caused by aPL in vitro*.*

### miR-146a-5p is enriched in hucMSC-exos and deliver to HTR8/SVneo cells to suppress the TRAF6 by binding to TRAF6 3'-untranslated regions

**Fig. 3:**
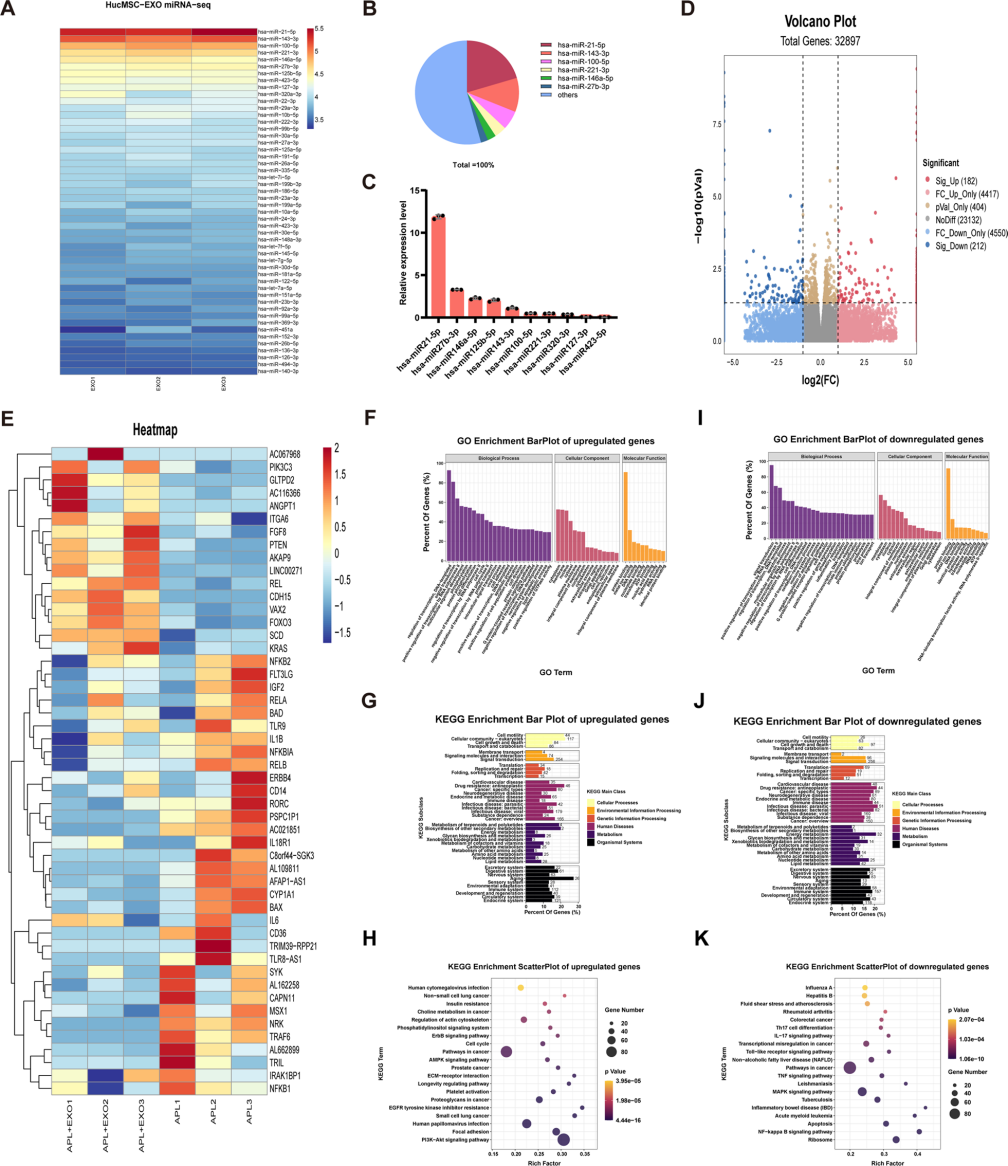
MiRNA sequencing analysis of hucMSC-exos and comprehensive analysis of genes and pathways involved in hucMSC-exos treatment based on mRNA high-throughput sequencing. **A** Heatmap of the top 50 most-enriched miRNAs expressed in hucMSC-exos (n = 3). **B** Relative percentage of miRNAs in total miRNA reads. **C** qRT-PCR analyzed the expression levels of the top 10 most-enriched miRNAs in hucMSC**-**exos (n=3). **D** Volcano plot of RNA-seq transcriptome data displaying the pattern of the gene expression profile in the HTR8/SVneo cells with or without hucMSC-exos treatment. The aPL + EXO group was incubated with hucMSC-exos (100 ug/ml) for 24 h post aPL stimulation (200 ug/ml). Red and blue dots indicate the significantly up- or down-regulated genes, respectively. p < 0.05, |log2FC|> 1. **E** Representative heatmap of differentially expressed genes between the aPL + EXO group and aPL group based on the above mRNA-seq data. (Red, relatively upregulated expression; blue, relatively downregulated expression). Each column represents one individual sample, and each row represents one single gene (n = 3). **F-K** Gene Ontology and Kyoto Encyclopedia of Genes and Genomes enrichment analysis of the up- and down-regulated genes in HTR8/SVneo cells of aPL + EXO group compare with aPL group. Gene percentage refers to the percentage of genes that were significantly enriched in the corresponding secondary class. Rich factor indicates the up-regulated and down-regulated expressed genes divided by the total number of genes. The smaller the p value, the higher enrichment degree. The diameter of the dots indicate the number of genes enriched in the corresponding signal pathways

**Fig. 4:**
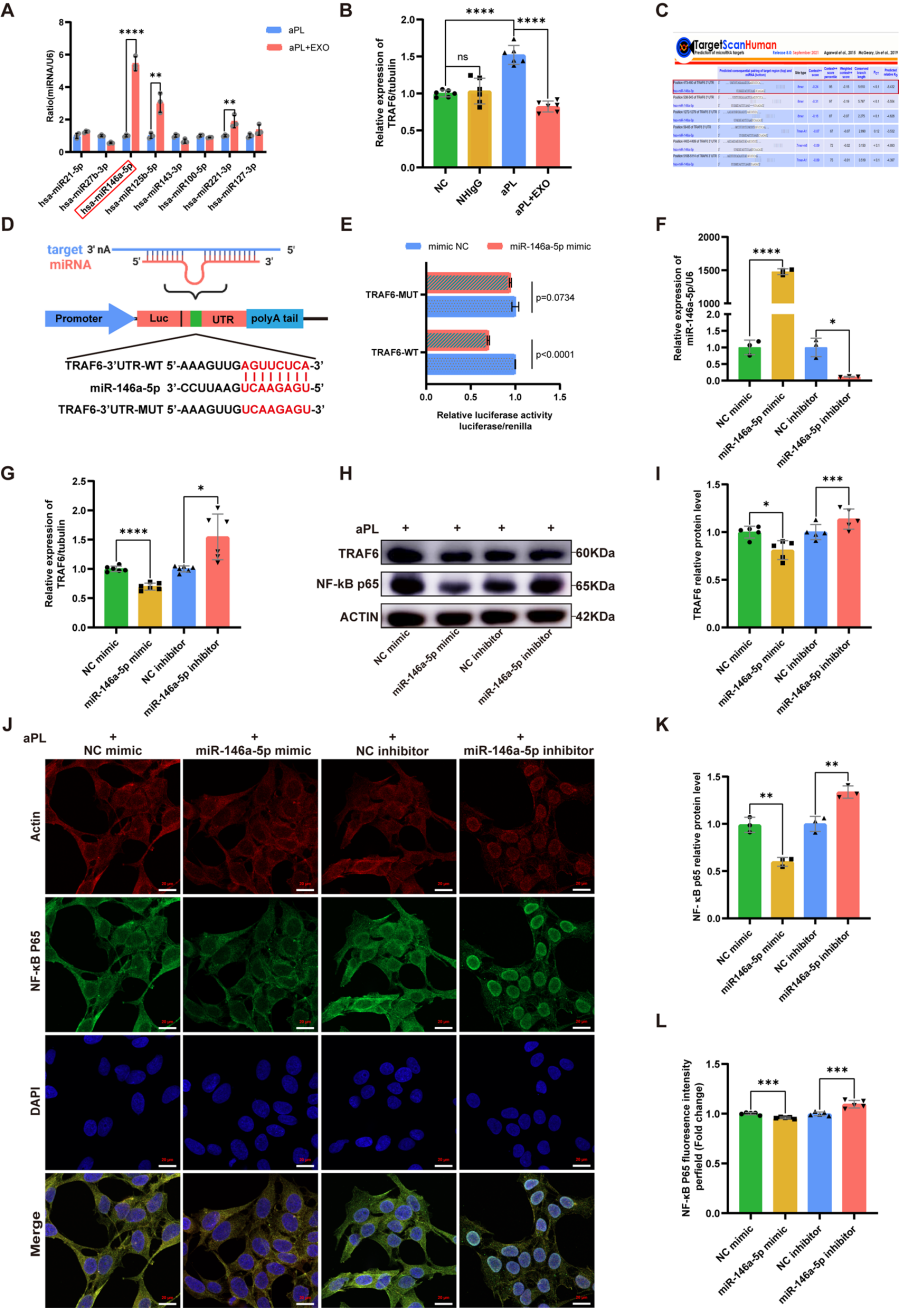
HucMSC-exos upregulated the miR-146a-5p expression which targets the TRAF6 and NF-κB signaling pathway in vitro. **A** Relative expression level of miRNAs analyzed by qRT‒PCR in the HTR8/SVneo cells in the aPL group and aPL + EXO group (the red box represent the HTR8/SVneo cells treated with aPL + EXO, the blue box represent the HTR8/SVneo cells treated with aPL) (n = 3). **B** Relative mRNA expression level of TRAF6 in the HTR8/SVneo cells analyzed by qRT‒PCR (n = 6). **C** StarBase analysis of the target genes of miR-146a-5p. **D** Schematic diagram depicted the predicted binding site of miR-146a-5p targeting the 3'-UTR of TRAF6. **E** Luciferase reporter gene assay of miR-146a-5p mimic treated HEK293T cells, which overexpressed either TRAF6-wildtype 3'UTR or TRAF6 -mutant 3'UTR (n = 3). **F**-**L** HTR8/SVneo cells were transfected with miR-146a-5p mimic or inhibitor before the aPL-injury process. **F** The relative mRNA expression level of miR-146a-5p in HTR8/SVneo cells transfected with miR146a-5p mimic (NC mimic) or inhibitor (NC inhibitor) were detected by qRT-PCR (n = 3). **G** The relative mRNA expression level of TRAF6 in HTR8/SVneo cells transfected with miR146a-5p mimic (NC mimic) or inhibitor (NC inhibitor) was detected by qRT-qPCR (n = 6). **H**, **I** and** K** Western blot analyzed the relative protein levels of TRAF6 and NF-κB p65 in HTR8/SVneo cells transfected with miR146a-5p mimic (NC mimic) or inhibitor (NC inhibitor), quantified by signal intensity normalized to ACTIN (n = 5). **J** and **L** Immunofluorescence (IF) analyzed the nuclear translocation of NF-κB p65 in the HTR8/SVneo cells transfected with miR-146a-5p mimic (NC mimic) or miR-146a-5p inhibitor (NC inhibitor), (NF-κB p65, green; Actin, red; DAPI nulcear stain, blue; Scale bars, 20 µm), (n = 5). *p < 0.05, **p < 0.01, ***p < 0.001, ****p < 0.0001

To identify the mechanism underlying the effects of hucMSC-exos, miRNA expression profiles were screened using microarray chip screening since exosomal miRNAs were reported to be vital in intercellular communication. As shown in Fig. 3A and B, miR-21-5p, miR-143-3p, miR-100-5p, miR-221-3p, and miR-146a-5p collectively accounted for nearly 43.4% of the total miRNA readings. According to qRT-PCR analysis (Fig. 3C), miR-21-5p, miR-27b-3p, and miR-146a-5p exhibited the highest levels of enrichment among the miRNAs present in hucMSC-exos. Furthermore, we conducted high-throughput mRNA sequencing to examine alterations in the mRNA expression profile triggered by hucMSC-exos in HTR8/SVneo cells treated with aPL at the genomic level (Fig. 3D). A total of 32897 genes were identified, with 394 showing differential expression (182 upregulated and 212 downregulated) in the aPL + EXO group when compared to the aPL group. In addition, gene ontology (GO) and Kyoto Encyclopedia of Genes and Genomes (KEGG) enrichment analyses were used to analyze the upregulated and downregulated genes, respectively. As shown in Fig. 3F and I, GO is mainly used to analyze major biological processes and molecular functions. Among the upregulated genes, the top 20 significantly enriched pathways (Fig. 3G and H) included the AMPK, cell cycle, ErbB, and PI3K-Akt signaling pathways, amongst others. Similarly, among the downregulated genes, the top 20 significantly enriched pathways included the toll-like receptor, MAPK, NF-κB, and apoptosis signaling pathways (Fig. 3J and K). Consistent with the above findings, the expression levels of the overwhelming majority of regulators, such as TRAF6, NRK, IRAK1, NF-κB, and BAX in the HTR8/SVneo cells were significantly decreased upon hucMSC-exos treatment (Fig. 3E). Additionally, the levels of the top enriched eight miRNAs were assessed in HTR8/SVneo cells following treatment with hucMSC-exos and we found that the miR-146a-5p was increased significantly (Fig. 4A). As mentioned in previous studies, miR-146a-5p is abundant in hucMSC-exos and has been demonstrated to participate in anti-inflammatory and tissue repair processes [42, 47–49]. Consistent with the mRNA sequencing results, the mRNA expression of TRAF6 in the aPL group was considerably higher than that in the NC group; nevertheless, this trend was significantly inhibited by hucMSC-exos (Fig. 4B).

To verify the potential mechanism of miR-146a-5p in hucMSC-exos-mediated repair effect of aPL-induced HTR8/SVneo cell injury, we concentrated on the downstream signaling pathway of miR-146a-5p. Using the TargetScan online database, we predicted the assumed targets of miR-146a-5p, as a previous study demonstrated its ability to diminish inflammation through specifically targeting TRAF6 [50]. Overlap analysis showed that TRAF6 is a potential target of miR-146a-5p (Fig. 4C). The direct relationship between miR-146a-5p and TRAF6 was confirmed through a double luciferase reporter gene assay. The luciferase activity remarkably diminished in HEK293T cells transfected with miR-146a-5p mimic and the TRAF6 wild-type (TRAF6-WT) reporter plasmids than that transfected with the TRAF6 mutant (TRAF6-MUT) reporter plasmids (Fig. 4D and E). All these findings imply that exosomal miR-146a-5p can bind the TRAF6 3'-untranslated regions (UTR) and suppress TRAF6 expression via post-translational repression.

### Exosomal miR-146a-5p ameliorates aPL-induced impaired cell function in vitro by targeting the TRAF6-mediated NF-κB signaling pathway

To further elucidate the regulatory relationship between exosomal miR-146a-5p and TRAF6, we established an in vitro model of cell injury induced by aPL. Briefly, HTR8/SVneo cells were transfected with the specific mimic or inhibitor of miR-146a-5p for 24 h, and then were exposed to aPL for 24 h. The expression of miR-146a-5p significantly increased in the group treated with miR-146a-5p mimic and markedly decreased in the miR-146a-5p inhibitor group, as determined by qRT-PCR analysis (Fig. 4F). Furthermore, the miR-146a-5p mimic significantly suppressed the mRNA expression of TRAF6, as observed in Fig. 4G, the expression level of TRAF6 in the inhibitor group was higher than that in the control group. Notably, TRAF6 facilitates the activation of NF-κB in numerous biological and pathological occurrences, encompassing inflammation, autophagy, and autoimmune diseases [51, 52]. We then examined the protein levels of TRAF6 and its downstream NF-κB p65 in HTR8/SVneo cells transfected with miR-146a-5p mimic or inhibitor. According to the western blot analysis, miR-146a-5p mimic significantly decreased the protein levels of TRAF6 and NF-κB p65, whereas which was increased in the miR-146a-5p inhibitor group (Fig. 4H, I and K). Moreover, immunofluorescence staining indicated that the miR-146a-5p mimic inhibited the aPL-induced movement of NF-κB p65 into the nucleus in HTR8/SVneo cells, conversely, the miR-146a-5p inhibitor promoted this process as shown in Fig. 4J and L.

**Fig. 5:**
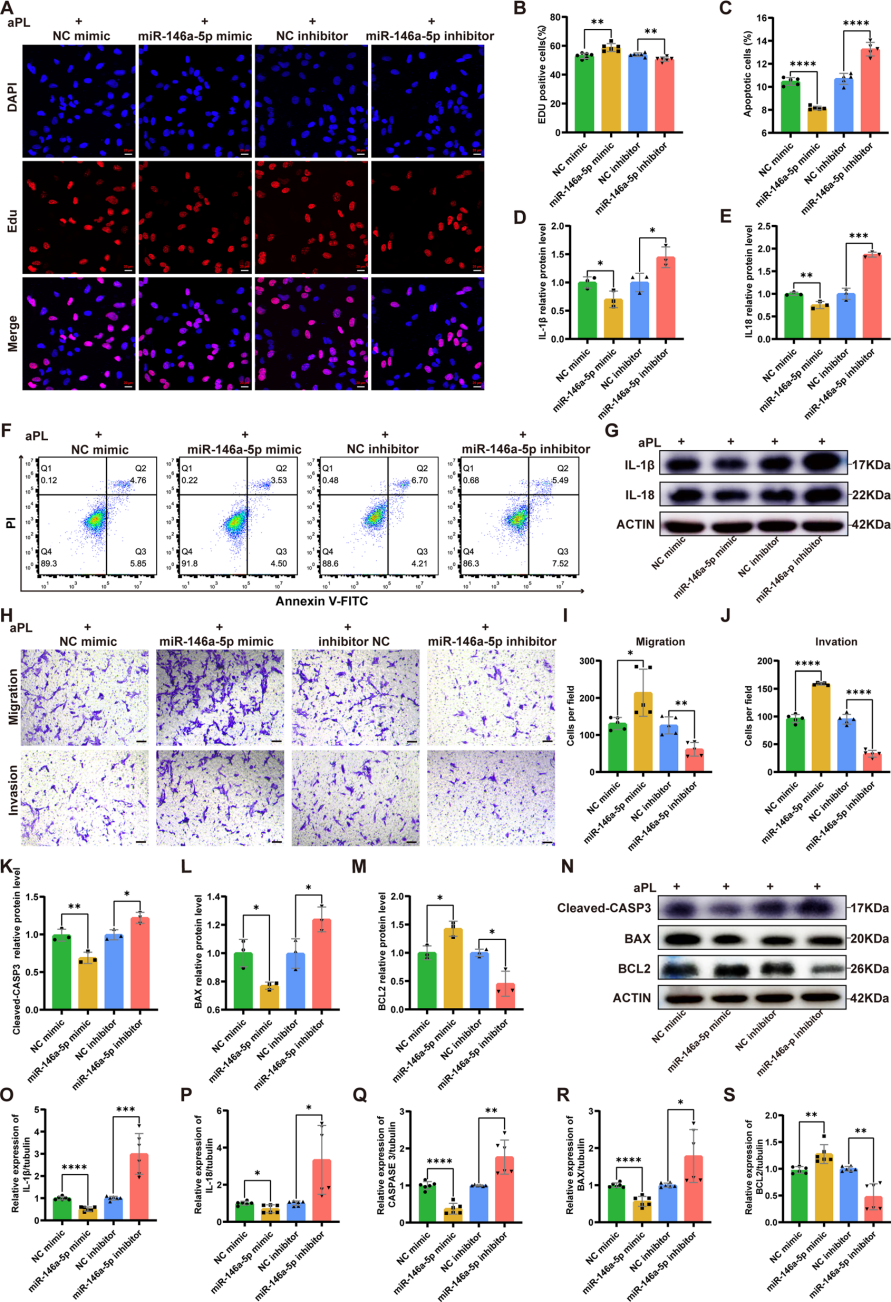
MiR-146a-5p originated from hucMSC-exos could ameliorate the HTR8/SVneo cells injury induced by aPL through inhibiting TRAF6-mediated NF-κB signaling pathway. **A**-**S** HTR8/SVneo cells were transfected with miR-146a-5p mimic or inhibitor before the aPL-injury process. **A** and **B** The proliferation ability of HTR8/SVneo cells transfected with miR-146a-5p mimic (NC mimic) or miR-146a-5p inhibitor (NC inhibitor) was detected by EdU assay (n = 6). Scale bars, 20 μm. **C** and **F** The cell apoptotic rate was determined by flow cytometry assay with annexin-V/PI staining (n = 5). **D**, **E** and **G** Western blot analyzed the relative protein levels of inflammation-associated (IL-1β, IL-18) in HTR8/SVneo cells, quantified by signal intensity normalized to ACTIN (n = 3). **H-J** Transwell experiment detected the abilities of migration and invasion of HTR8/SVneo cells transfected with miR-146a-5p mimic (NC mimic) or inhibitor (NC inhibitor) (n = 5). Scale bars, 100 μm. **K-N** Western blot analyzed the relative protein levels of apoptosis-related (Cleaved-CASP3, BAX and BCL2) in HTR8/SVneo cells transfected with miR-146a-5p mimic (NC mimic) or miR-146a-5p inhibitor (NC inhibitor), quantified by signal intensity normalized to ACTIN (n = 3). **O-S** The mRNA expression levels of inflammation-associated (IL-1β, IL-18) and apoptosis-related (CASP3, BAX and BCL2) in the HTR8/SVneo cells transfected with miR-146a-5p mimic (NC mimic) or miR-146a-5p inhibitor (NC inhibitor) were analysed by qRT‒PCR (n = 6). *p < 0.05, **p < 0.01, ***p < 0.001, ****p < 0.0001

Functional experiments were conducted to verify the repair effect of exosomal miR-146a-5p on aPL-induced functional damage in HTR8/SVneo cells. EdU assay demonstrated that cells transfected with the miR-146a-5p mimic showed higher proliferation ability compared to those transfected with the NC mimic; however, the miR-146a-5p inhibitor group had lower proliferation ability in comparison to the NC inhibitor group (Fig. 5A and B). Moreover, the apoptotic rate in the miR-146a-5p mimic group was lower than that in the NC mimic group; however, the miR-146a-5p inhibitor group showed a higher apoptotic rate than the NC inhibitor group, as determined using flow cytometry (Fig. 5C and F). HTR8/SVneo cells transfected with the miR-146a-5p mimic showed stronger migration and invasion abilities compared to cells transfected with the NC mimic; conversely, these abilities were further suppressed in HTR8/SVneo cells transfected with the miR-146a-5p inhibitor, as opposed to those transfected with the NC inhibitor. Additionally, we detected the protein levels of the TRAF6/NF-κB axis downstream, which play a role in both inflammation and apoptosis pathways. As shown in Fig. 5D, E and G, transfection of the miR-146a-5p mimic remarkably reduced the expression of inflammation-associated proteins, specifically IL-1β and IL-18, which were elevated in the miR-146a-5p inhibitor group. Furthermore, the miR-146a-5p mimic group exhibited significant reductions in levels of pro-apoptotic proteins (Cleaved-CASP3 and BAX), while the level of anti-apoptotic protein BCL2 was increased. Conversely, the miR-146a-5p inhibitor group displayed the opposite trend of these apoptosis-related proteins (Fig. 5K-N). The qRT-PCR results for the transcriptional levels of factors associated to inflammation and apoptosis were consistent with the findings from the western blot assay (Fig. 5O-S).

These findings demonstrate the irreplaceable function of exosomal miR-146a-5p in the downregulation of TRAF6 and NF-κB signaling pathway in aPL-induced injury in trophoblasts.

### TRAF6 knockdown inhibited the activation and nuclear translocation of NF-κB p65 and attenuated the aPL-induced injury in HTR8/SVneo cells

**Fig. 6:**
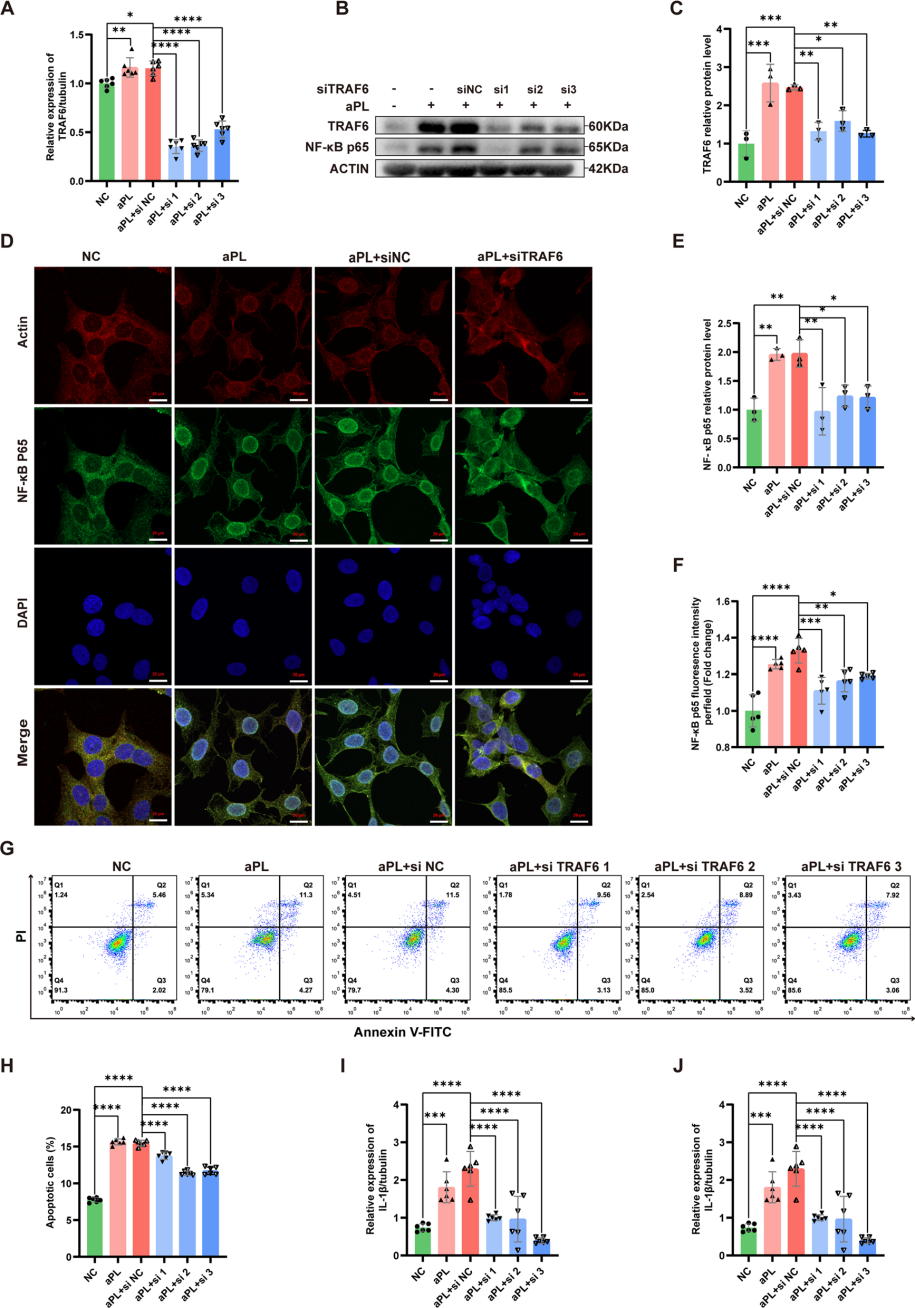
Knocking down of TRAF6 could inhibit the nuclear translocation of NF-κB p65 and attenuate the aPL-induced HTR8/SVneo cells injury. **A** The mRNA expression level of TRAF6 was detected by qRT-PCR in HTR8/SVneo cells infected with TRAF6 siRNA (n = 6). **B**, **C** and **E** The relative protein levels of TRAF6 and NF-κB p65 were detected by western blot in HTR8/SVneo cells infected with TRAF6 siRNA, quantified by signal intensity normalized to ACTIN (n = 3). **D** and **F** IF analyzed the nuclear translocation of NF-κB p65 in HTR8/SVneo cells infected with TRAF6 siRNA (NF-κB p65, green; Actin, red; DAPI nulcear stain, blue), (n = 5). Scale bars, 20 µm. **G** and **H** The cell apoptotic rate was determined by flow cytometry analysis with annexin-V/PI staining in HTR8/SVneo cells infected with TRAF6 siRNA (n = 5). **I** and **J** The mRNA expression levels of inflammatory cytokine factors (IL-1β and IL-18) in HTR8/SVneo cells infected with TRAF6 siRNA were examined by qRT-PCR assay (n = 6). *p < 0.05, **p < 0.01, ***p < 0.001, ****p < 0.0001

Based on the aforementioned findings, it has been verified that exosomal miR-146a-5p effectively targetes and suppress TRAF6, thereby alleviating cell injury induced by aPL. To further clarify the regulatory role of TRAF6 on the NF-κB signaling pathway, we utilized siRNA to silence TRAF6 in HTR8/SVneo cells pretreated with aPL. According to qRT-PCR analysis, the mRNA level of TRAF6 were found to be increased in aPL-pretreated HTR8/SVneo cells, which was consistent with the previous findings; however, the expression of TRAF6 was notably reduced in HTR8/SVneo cells transfected with siTRAF6—all of the three target siRNAs had expected inhibition efficiencies (Fig. 6A). Western blot validated that siTRAF6 could block the aPL-induced upregulation of TRAF6 and activation of NF-κB in HTR8/SVneo cells (Fig. 6B, C and E). Immunofluorescence staining revealed that the high fluorescence intensity and obvious nuclear localization of NF-κB p65 in HTR8/SVneo cells injured by aPL were reduced upon TRAF6 knockdown (Fig. 6D and F). Furthermore, we measured the apoptotic rate of HTR8/SVneo cells in different groups using flow cytometry (Fig. 6G and H). The high apoptotic rate of HTR8/SVneo cells injured by aPL was reversed by TRAF6 knockdown. In addition, the mRNA levels of inflammatory factors (IL-1β and IL-18) in HTR8/SVneo cells showed a notable decrease due to TRAF6 knockdown detected by qRT-PCR (Fig. 6I and J). These findings suggest that TRAF6 knockdown can improve impaired cellular function and inhibit apoptosis and inflammation in HTR8/SVneo cells after aPL injury.

### HucMSC-exos prevented fetal resorption and attenuated placental dysfunction in mice with antiphospholipid antibody syndrome

**Fig. 7:**
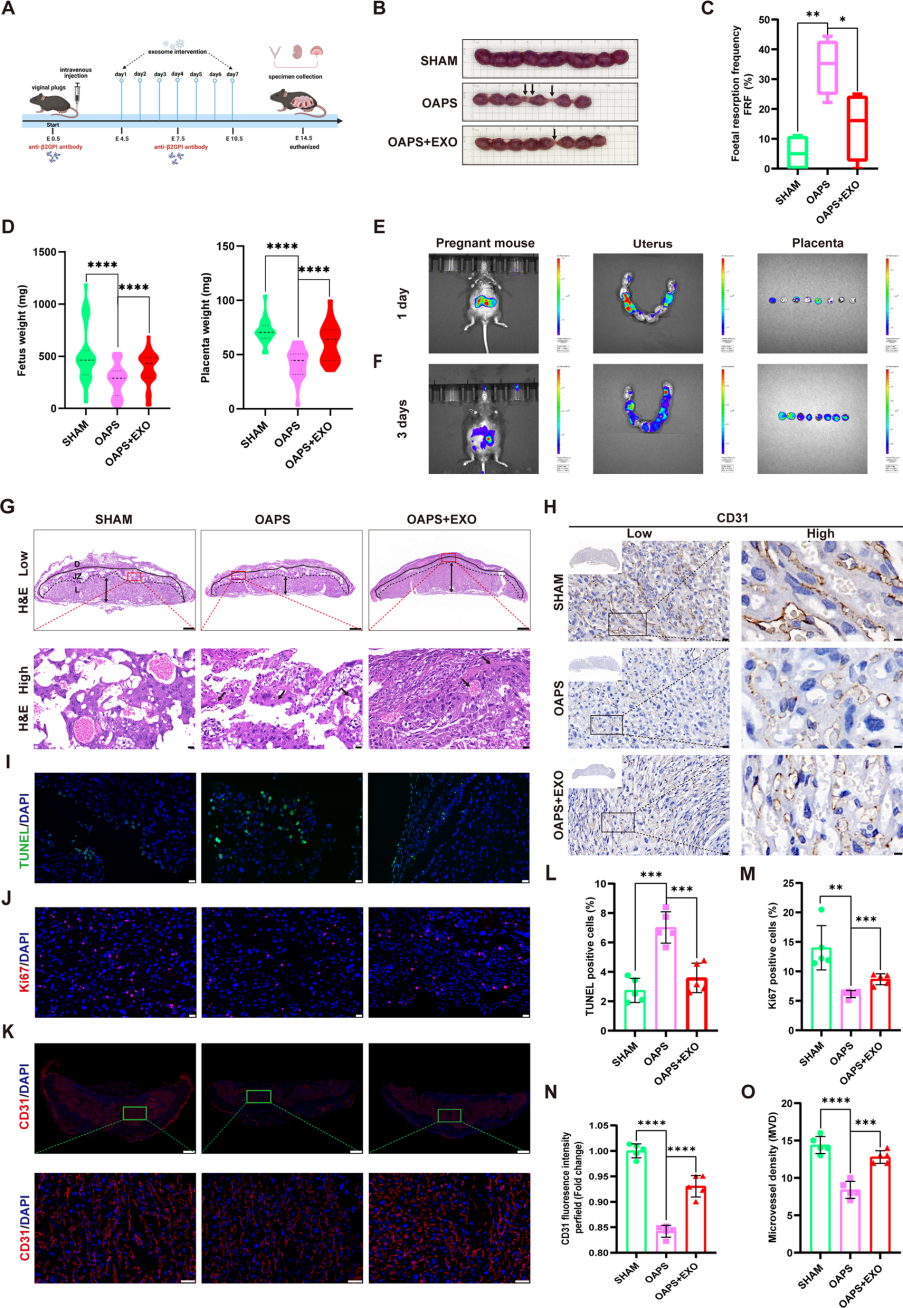
HucMSC-exos attenuated placental dysfunction in mice with antiphospholipid antibody syndrome. **A** Schematic illustration of OAPS mice model and therapeutic experiments. **B** Representative photographs of uterus morphology on E14.5, arrows represent the fetal absorption. **C** Fetal resorption frequency was assessed at E14.5. **D** Statistical analysis of fetal and placental weight in the three groups of mice. **E** and** F** In vivo imaging showed the DIR-labelled hucMSC-exos can be traced in the uters and placentas after 1 day and 3 consecutive days of tail vein injection. **G** Representative low- and high-magnification images of H&E-stained cross sections of placentas, the arrows point out the necrosis area and diseased blood vessel (decidua basalis (D); junctional zone (JZ); labyrinth (L)). Scale bars, 500 µm and 20 µm. **H** and **O** Representative low- and high-magnification images of IHC of CD31 and the microvessel density statistics of placental blood vessels (n = 5). Scale bars, 20 µm and 5 µm. **I** and **L** Representative images of TUNEL assay showing the apoptotic cells of mice placenta (TUNEL, green; DAPI nuclear stain, blue), quantified by the proportion of TUNEL^+^ cells (n = 5). Scale bars, 50 μm. **J** and **M** Representative images of Ki67 assay showing the proliferative cells of mice placenta (Ki67, red; DAPI nuclear stain, blue), quantified by the proportion of Ki67^+^ cells (n = 5). Scale bars, 50 μm. **K** and** N** Immunofluorescence of CD31 in mice placenta (CD31, red; DAPI nuclear stain, blue) and quantified by fluorescence intensity (n = 5). Scale bars, 50 μm. *p < 0.05, **p < 0.01, ***p < 0.001, ****p < 0.0001

To assess the protective effect of hucMSC-exos against aPL-induced placental dysfunction in vivo, the OAPS mouse model was established by an intravenous injection of anti-β2GPI antibody developed by Affinity Biosciences as previously described [53], and an effective dose of hucMSC-exos was injected into the tail vein (Fig. 7A). Additionally, the fetal resorption frequency (FRF) was assessed at E14.5. Animal models formed from prepared antibodies had higher FRF and were more stable than those formed from patient serum antibodies [54]. Additionally, the OAPS mice exhibited higher FRF than the sham mice, indicating the successful construction of the OAPS mouse model (Fig. 7B and C); the FRF was significantly decreased by hucMSC-exos as expected. In line with our previous results [53], the weights of the fetus and placentas in OAPS mice were notably lesser than those in sham mice; however, the adverse effect was effectively reversed by hucMSC-exos (Fig. 7D). Furthermore, in vivo imaging showed that the DiR-labeled exosomes could be traced in the uterus and placentas one day after intravenous injection, and the fluorescence signal was stronger after three consecutive days of injection (Fig. 7E and F). Compared with the sham mice, significant necrotic lesions appearing as dots or ribbons in the hematoxylin and eosin in the H&E placental slices of the OAPS mice, with or without degeneration and shedding of vascular wall cells, while hucMSC-exos improved these pathological phenomena (Fig. 7G). TUNEL (Fig. 7I and L) and Ki67 (Fig. 7J and M) assays revealed increased cell apoptotic rate and decreased cell proliferative rate in OAPS mice placentas; hucMSC-exos significantly reversed this phenomenon. Furthermore, we performed immunofluorescence and immunohistochemistry staining of CD31 (Fig. 7K and H) in mouse placentas to evaluate the placental blood supply; hucMSC-exos obviously improved the reduction of CD31 fluorescence intensity (Fig. 7N) and micro-vessel density (MVD) in OAPS mice (Fig. 7O). These results confirm that the anti-β2GPI antibody harmed the function of the trophoblast cells and the placenta, which was alleviated after hucMSC-exos treatment consistent with the in vitro experiments. These findings highlight the critical therapeutic role of hucMSC-exos in placental dysfunction in OAPS mice by alleviating pathological reactions and promoting placental development and vascular remodeling.

### HucMSC-exos enhanced the expression of miR-146a-5p targeting the TRAF6-mediated NF-κB signaling pathway in vivo

**Fig. 8:**
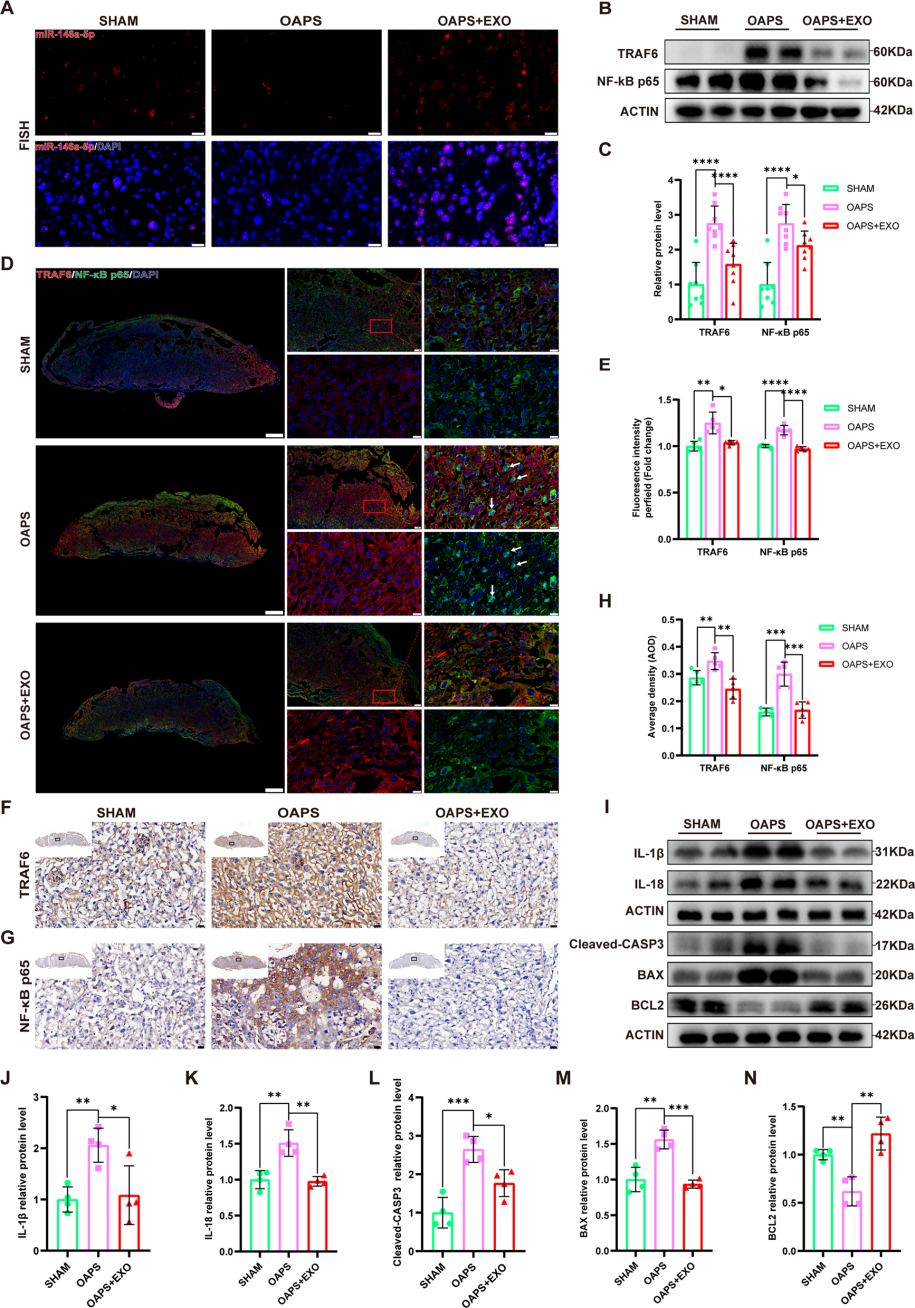
HucMSC-exos alleviated the placental dysfunction in OAPS mice by delivering miR-146a-5p targeting the TRAF6/ NF-κB axis. **A** Representative images of FISH of miR-146a-5p in mice placenta sections. Scale bars, 20 µm. **B** and **C** Western blot analyzed the relative protein levels of TRAF6 and NF-κB p65 in mice palcenta, quantified by signal intensity normalized to ACTIN (n = 8). **D** and **E** Representative low- and high-magnification images of TRAF6 and NF-κB p65 IF staining in mice placenta (TRAF6, red; NF-κB, green; DAPI nuclear stain, blue), quantified by fluorescence intensity (n = 5). Scale bars, 500 μm and 20 μm. **F**-**H** Representative low- and high-magnification images of immunohistochemistry of TRAF6 and NF-κB p65 in mice placenta, quantified by average density (n = 5). Scale bars, 500 μm and 20 μm. **I**-**N** Western blot analyzed the relative protein levels of inflammation-associated (IL-1β, IL-18) and apoptosis-related (Cleaved-CASP3, BAX and BCL2) proteins in mice palcenta, quantified by signal intensity normalized to ACTIN (n = 4). *p < 0.05, **p < 0.01, ***p < 0.001, ****p < 0.0001

The above experiments revealed that hucMSC-exos could enter the uterus and placentas of pregnant mice. To investigate the involvement of miR-146a-5p in the hucMSC-exos-mediated restoration of placental dysfunction in vivo, fluorescence in situ hybridization (FISH) was conducted to detect the expression of exosomal miR-146a-5p (Fig. 8A). The exosome intervention group showed a higher level of miR-146a-5p than the OAPS group. The western blot (Fig. 8B and C) and immunohistochemistry (Fig. 8F-H) reaveled that hucMSC-exos significantly reversed the increased expression of TRAF6 and NF-κB p65 in the placenta of OAPS mice. Additionally, immunofluorescence staining revealed stronger fluorescence intensity of TRAF6 and more nuclear localization of NF-κB p65 in the OAPS mice than in the sham mice; this trend was significantly  suppressed by hucMSC-exos (Fig. 8D and E). Furthermore, the downstream molecules of the TRAF6/NF-κB axis, including inflammation-associated (IL-1β, IL-18) and apoptosis-related (Cleaved-CASP3, BAX, BCL-2) proteins were markedly reversed by hucMSC-exos (Figs. 8I-N). These results indicate that hucMSC-exos effectively attenuated placental dysfunction in OAPS mice via the miR-146a-5p/TRAF6/NF-κB pathway in vivo.

## Discussion

Although the potential mechanisms underlying OAPS have been extensively researched, developing effective therapeutics to overcome aPL-induced OAPS remains challenging. Previous studies on animal models of multiple diseases and human patients have proven the benefits of hucMSC-exos [25–39]. However, the mechanism underlying the therapeutic potential of hucMSC-exos in OAPS remains unclear. Our study provides the first hand evidence for the potential molecular mechanism by which hucMSC-exos improves aPL-induced trophoblast injury and placental dysfunction in OAPS by transmitting miR-146a-5p.

Current researches indicate that aPLs, particularly the anti-β2GPI antibody, can impact the migration, invasion, and proliferation abilities of trophoblasts, leading to shallow placental implantation and dysfunction, ultimately causing placental-mediated pregnancy complications, including recurrent miscarriage, pre-eclampsia or stillbirth [6, 55, 56]. Previous studies have demonstrated that the TLR4/MyD88 pathway was involved in trophoblast injury and inflammasome activation mediated by anti-β2GPI antibody [9, 57, 58]. Furthermore, the low-density lipoprotein receptor apoER2 has also been shown to be associated with aPL-induced trophoblast dysfunction [59, 60]. In our study, HTR8/SVneo cells were exposed to aPL extracted from patients with OAPS in vitro, resulting in HTR8/SVneo cell injury. HucMSC-exos significantly ameliorated the aPL-induced impaired migration, invasion, and proliferation abilities in HTR8/SVneo cells. Additionally, hucMSC-exos inhibited cell apoptosis (evidenced by increased BCL2 and decreased Cleaved-CASP3, BAX levels) and inflammation (evidenced by decreased IL-18, IL-1β). These results prove that hucMSC-exos are a good source of OAPS protection during acellular therapy.

Many researches have demonstrated that hucMSC-exos possess the ability to alleviate various diseases by delivering specific miRNAs to target cells [61–64]. In present study, high-throughput microarray analysis revealed a substantial presencee of miR-146a-5p in hucMSC-exos, and subsequent qRT-PCR analysis showed an elevation in the expression level of miR-146a-5p in HTR8/SVneo cells following treatment with hucMSC-exos. Moreover, earlier studies have reported the importance of miR-146a-5p in tissue repair and anti-inflammatory [47–50, 65]. Therefore, we selected miR-146a-5p originating from hucMSC-exos as the key molecule for hucMSC-exos-mediated improvement of aPL-induced HTR8/SVneo cell injury. Furthermore, we found that miR-146a-5p inhibitor significantly diminish the therapeutic effect of the miR-146a-5p mimic on aPL-induced cell injury, verifying the potential of exosomal miR-146a-5p in trophoblasts.

Moreover, we confirmed TRAF6 as the direct target gene of exosomal miR-146a-5p using mRNA sequencing, qRT-PCR assay, and online databases. This confirmation was verified by luciferase reporter assay, western blot analysis, and a range of functional experiments. TRAF6 has been recognized as a key regulatory factor responsible for various biological processes [52]. According to Lu et al. [66], TRAF6 exhibited high expression in spinal astrocytes and contributed to the exacerbation neuropathic pain through promoting TNF-α and IL-1β signaling conduction. Furthermore, studies have reported that the upregulated TRAF6 can inhibite autophagy and promote microglia pyroptosis in inflammatory pain [50]. Min Y et al. [51] discovered that suppression of TRAF6 expression could reverse autophagy activation by inhibiting the NF-κB signaling pathway. To comprehend the intrinsic regulatory function of TRAF6 in signal transduction, we utilized siTRAF6 to knock down TRAF6 in HTR8/SVneo cells pretreated with aPL in present study. Suppression of the NF-κB signaling pathway and inhibition of aPL-induced cell injury were observed upon TRAF6 knockdown. These findings confirmed that miR-146a-5p, originating from hucMSC-exos, significantly ameliorated aPL-induced trophoblast injury by suppressing the TRAF6-mediated NF-κB axis in vitro.

Many studies have revealed the benefits of hucMSC-exos in inhibiting trophoblasts apoptosis in the placental tissue of rats with pre-eclampsia [37, 38, 67]. Nevertheless, there is currently no existing study has documented the therapeutic effects of hucMSC-exos in OAPS in vivo. According to a recent study, the intravenous method was found to be the most commonly utilized approach for administering exosomes to treat diseases in vivo [68]. Therefore, we opted for the route of drug delivery through tail vein injection for exosomes. In the OAPS mouse model, multiple tail vein injections of hucMSC-exos resulted in a notable decrease fetal absorption and improvement in placental dysfunction. The placenta is an important organ that plays an unsubstitutable affect in the health and well-being of the fetus and its mother [69, 70]. Placental growth is a meticulously controlled an precisely coordinated procedure [71].

Therefore, normal cell proliferation and apoptosis are essential for the proper development of the placenta. Our research indicates that hucMSC-exos effectively supressed cell apoptosis and stimulated the proliferation of placental cells in OAPS mice. Furthermore, we investigated the impact of hucMSC-exos on angiogenesis in OAPS mice; and the elevated quantities of CD31 and MVD indicated that hucMSC-exos attenuated placental dysfunction in OAPS mice by alleviating vascular remodeling. Our findings provide further in vivo evidence that hucMSC-exos may potentially serve as an alternative method for treating OAPS.

Furthermore, hucMSC-exos effectively prevented apoptosis and inflammation in placentas of OAPS mice by restoring BCL2 and inhibiting Cleaved-CASP3, BAX, IL-1β and IL-18. OAPS mice exhibited elevated levels of TRAF6 and NF-κB p65 than the sham mice; but the administration of hucMSC-exos suppressed these alterations. Earlier research have indicated that the levels of miR-146a-5p in the placenta of individuals experiencing premature pregnancy and recurrent miscarriage are lower than those in the placenta of normal individuals [72, 73]. Moreover, Yang C et al. [47] provided evidence that miR-146a-5p originating from amniotic fluid mesenchymal stem cells exerted an anti-inflammatory effect on human trophoblastic cells. In previous studies, it has been noted by scholars that miR-146a-5p has the ability to improve the survival of embryos in cases of unexplained recurrent spontaneous miscarriage by encouraing the trasnsformation of decidual macrophage into M2 phenotype [74]. In our own investigation, the level of miR-146a-5p was decreased in OAPS mice than in the control subjects; however this situation was reversed by hucMSC-exos. According to our study, hucMSC-exos can suppress cell death and reduce inflammation in the placental tissue of OAPS mice by delivering miR146a-5p that blocks the TRAF6/NF-κB axis, thereby improving placental function damage in OAPS mice. Encouragingly, we observed increased fluorescence intensity and more nuclear localization of NF-κB p65 in placental tissue of individuals with OAPS compared to that in healthy individuals (Additional file 1: Figure S2), provide novel evidence for the pathogenesis of OAPS and indicating potential strategy for its clinical treatment.

There are some limitations in our research. First, we could not rule out the potential effects of other miRNAs from hucMSC-exos; therefore, further clarification is needed. Second, we used loss/acquisition of function through specific siRNA, mimic, and inhibitor transfections to estimate the therapeutic effects of hucMSC-exos. Gene-knockout animal models may provide more objective and useful information for further studies of the mechanisms of OAPS. Exosomes have enormous potential as desirable drug/gene delivery vectors in the field of regenerative medicine [64]. Future studies will have significant implications for modifying and engineering exosomes with bioactive materials for better targeting, thereby optimizing their therapeutic effects on OAPS.

## Conclusions

In summary, our study systematically demonstrated the therapeutic potential of hucMSC-exos in OAPS in vivo and in vitro. We discovered that hucMSC-exos have the ability to transport miR-146a-5p into HTR8/SVneo cells and mouse placenta, block the TRAF6/NF-κB signaling pathway, and ultimately ameliorate trophoblast injury and placental dysfunction induced by aPL. These findings offer insights into the pathogenesis of OAPS and establish a new platform for designing therapeutic strategies.

## Materials and methods

### Patient inclusion criteria and sample collection

A total of 25 patients with OAPS who met Sydney criteria [1] and visited the obstetrics department of Shandong Provincial Hospital Affiliated to Shandong First University were incorporated into our study. Patients with infections, inflammatory disorders or malignancies were excluded. Gestational age-matched pregnant women with negative aPLs, who had no related pregnancy complications and had at least one healthy pregnancy, were considered as normal healthy controls. The details of all the recruited participants are presented in Additional file 1: Table S1. Peripheral venous blood samples from both normal healthy controls and patients with OAPS were collected in vacuum tubes without EDTA, left at a room temperature (RT) of 20–26 ℃ for at least 30 min, following centrifuged at 3000 × g for 15 min. Finally, the supernatant was collected gently and sterilized for subsequent experiments.

### Extraction, culture, and identification of hucMSCs

Three umbilical cords were collected from healthy newborns delivered via the cesarean section at the Shandong Provincial Hospital Affiliated to Shandong First Medical University. Huc-MSCs were extracted from human umbilical cord with the tissue explants adherent method. In brief, umbilical cords obtained under sterile conditions were cut into small pieces (2–3 cm) after removal of arteries and veins. These pieces were further minced to tiny chunks with a size of approximately 2–3 mm. Afterwards, the obtained pellets were suspended in DMEM/F12 (Gibco, Grand Island, NY, USA) culture medium with 10% FBS, and then seeded in a T25 flask. The cells are cultured at 37 °C in a CO2 incubator until they attain fusion.

The passage 3 (P3) hucMSCs were inoculated into a 12-well plate (1 × 10^4^ cells per well). Per the manufacturer’s instructions, 1 mL of adipogenic, osteogenic, or chondrogenic differentiation complete culture medium was added to each well (Haixing Biotechnology Co., LTD, Suzhou, China). After 3 weeks, the formation of fat droplets, calcium nodules, and cartilage components was observed under a microscope (Olympus, Japan).

HucMSCs were seeded in 96-well cell culture plates. Following fixation of the cells with 4% paraformaldehyde and subsequent washing with phosphate-buffered saline (PBS), they were then sealed using goat serum. Subsequently the cells were incubated overnight with primary antibodies CD105 and CD45 (1:100 dilution; Proteintech, San Diego, CA, USA). The next day, hucMSCs were washed thrice and then exposed to a secondary antibody (Alexa Fluor 594/488-conjugated goat anti-rabbit IgG, 1:100 dilution) (Boster, USA) under RT for 1 h in the dark. Next, after washing with PBS thrice, hucMSCs were incubated in DAPI solution (Boster, USA) for 8 min. Subsequently, the expression levels of CD105 and CD45 on the surfaces of hucMSC were observed using an inverted fluorescence microscope (Olympus, Japan).

The P3 hucMSCs were digested into a single-cell suspension, rinsed thrice with precooled PBS, and resuspended into 1.5 mL Eppendorf tubes with a concentration of 1 × 10^5^ cells/l00 μL. Next, the labeled antibodies (PE-CD105, FITC-CD90, PE-CD44, PE-CD34, FITC-CD45, and APC-HLA-DR), and their respective isotype control antibodies (Proteintech) were added into each Eppendorf tube and left to incubate for a duration of 20 min. Subsequently, the cells were rinsed thrice with 500 μL of PBS and suspended in 100 μL of PBS. Cell detection was performed using the Accuri C6 flow cytometer (BD Biosciences, San Jose, CA, USA) and data analysis was conduccted using the FlowJo software.

### Extraction and characterization of hucMSC-exos

The hucMSCs in the logarithmic growth phase, specifically P3–P5, were cultured for 48 h in the serum-free DMEM/F12 medium (Gibco). According to a previously established standardization principle for exosome isolation and identification, hucMSC-exos were extracted from the hucMSC supernatant using ultracentrifugation as illustrated in Additional file 1: Figure S1. This procedure has been peciously established [75, 76]. Furthermore, the morphology of the exosomes was examined through TEM (HT7700, Hitachi, Japan), while the size and concentration of particles were measured using NTA on a NanoSight (Xiuyue Biol, Jinan, China).

### Exosome labeling, internalization, and tracing

For the in vitro experiment, exosomes were labeled with PKH-67 (MIDI67, Midi Kit, Sigma-Aldrich**,** Burlington, MA, USA) as per the manufacturer’s recommendations. Fluorescent labeled hucMSC-exos (100 ug/mL) were added into the supernatant of HTR8/SVneo cells for 24 h. Following fixation in 4% paraformaldehyde at RT for 15 min, the cytoskeleton was marked using phalloidin-iFluor594 (ab176757, Abcam, Cambridge, United Kingdom) and the nuclei were stained with DAPI (Boster). The fluorescence signals were detected and photographed using a Zeiss CellDiscoverer 7 with ZEN 3.1 professional analysis software to ascertain whether the cells could take up the exosomes.

For in vivo visualization, hucMSC-exos were labeled with DiR (Life Technologies, Carlsbad, CA, USA). In brief, purified exosomes were incubated in the DiR solution (7.0 mL of 7.5 µM DiR dilution in PBS) for 20 min under RT in dark. DiR-labeled exosomes were then injected into the tail veins of pregnant mice. After one day and three consecutive days of exosomal intervention respectively, the two pregnant mice were continuously anesthetized with isoflurane, and images were obtained using the IVIS SpectrumCT In Vivo Imaging System (PerkinElmer, Waltham, MA, USA).

### Preparation of antiphospholipid antibody immunoglobulin G and anti-β2GPI antibody

aPL-IgG was purified from normal healthy controls and patients with APS using NAb™ Protein A Plus Spin Kit (Thermo Fisher Scientific). The collected sera were passed through a Protein A Agarose Column thrice according to the producer’s illustration. IgG purified from normal healthy controls and patients with APS was named NHIgG and aPL, respectively. The endotoxins in the purified IgG were detected using the Chromogenic Endotoxin Quantitation Kit (Thermo Fisher Scientific). Furthermore, anti-β2GPI antibody used for the animal experiment was developed in collaboration with Affinity Biosciences, as previously described [53]. Technical route of antibody preparation is shown in Additional file 1: Figure S3. In our experiment, aPL-IgG extracted from human serum was used for cell experiments in vitro, and the anti-β2GPI antibody was used for animal experiments in vivo.

### EdU assay

HTR8/SVneo cells were inoculated in the corning 3599 cell culture plate at a concentration of 8 × 10^3^ cells per well. After adhesion, cells were treated with NHIgG or aPL (200 µg/mL) for 24 h, and the exosome treatment group was incubated with hucMSC-exos (100 µg/mL) for 24 h after aPL treatment. The different processing groups were named as follows: NC, NHIgG, aPL, and aPL + EXO. To assess the cells’ ability to proliferate, the Beyoclick™ Edu Cell Proliferation Kit Alexa Fluor 647 (Beyotime, China) was employed. Cell proliferation was observed and analyzed using a Zeiss Cell Discoverer 7 equipped ZEN 3.1 professional analysis software.

### Apoptosis analysis

Apoptosis of HTR8/SVneo cells in the different intervention groups was detected using the Annexin V-FITC/PI Apoptosis Detection Kit (BD Biosciences) on an Accuri C6 flow cytometer (BD Biosciences). Moreover, in situ apoptosis in mouse placental sections was detected using a TUNEL Bright Green Apoptosis Detection Kit (A112-03, Vazyme, China), and fluorescence signals were observed using a NIKON digital sight DS-FI2 (Nikon, Japan). Apoptosis in placental tissue was illustrated by TUNEL positive cell ratios in five randomly selected fields of view.

### Cell migration and invasion assays

The migration ability of the HTR8/SVneo cells was detected in a 24-well Transwell cell culture plate (Corning, NY, USA). Cells from different groups were inoculated in the upper chamber diluted with 200 μL of the serum-free medium at a concentration of 5 × 10^4^ cells/well, and 600 μL of complete medium were supplemented to the lower chamber. After a 24 h incubation period, the cells were fixed and stained. Five fields of view were randomly selected to count the number of cells passing through the membrane under an optical microscope. For the invasion experiment, the upper chamber of the insert was coated with 60 μL of Matrigel (Sigma Aldrich) at a working concentration of 1 mg/mL before seeding cells.

### miRNA microarray chip screening and mRNA sequencing

Total RNA was extracted from hucMSC-exos for miRNA microarray chip screening, performed by LC-Bio Technology (Hangzhou, China). Additionally, Illumina Hiseq 2000/2500 was used to sequence the constructed library. Total RNA was extracted from the aPL and aPL + EXO groups using TRIzol (AG21101, Accurate Biotechnology, Hunan, China) for mRNA sequencing performed by LC-Bio Technology (Hangzhou, China).

### Dual-luciferase reporter gene assay

The targeting relationship between miR-146a-5p and TRAF6 and the connection site between miR-146a-5p and TRAF6 3’UTR were calculated using the targetscan software. The WT and MUT 3'UTR of TRAF6 were synthesized and linked to the luciferase reporter vector PHY-811 synthesized by Beijing Syngentech (Beijing, China). When the HEK293T cells with good growth status reached at approximately 80% cell fusion, cells were transfected with mixtures of TRAF6-WT or TRAF6-MUT plasmids with NC or miR-146a-5p mimic, respectively. After 24 h, a Dual-Luciferase Reporter Assay System (Promega, E1910, WI, USA) was used to detected the luciferase activity. All experiments were performed in triplicates.

### Quantitative real-time polymerase chain reaction (qRT-PCR)

Total RNA was extracted from exosomes using the miRNeasy Mini Kit (Qiagen), and miRNA first strand cDNA was synthesized using the miRNA 1st Strand cDNA Synthesis Kit (AG11716, Accurate Biotechnology). Additionally, RNA was extracted from cells using TRIzol (AG21101, Accurate Biotechnology), cDNA synthesis was carried out on the ABI 7500 Real-Time PCR system (Applied Biosystems, Waltham, MA, USA) using an Evo M-MLV Mix Kit containing gDNA Clean reagents (AG11734, Accurate Biotechnology). Amplification was then conducted on a LightCycler 480 II using an SYBR Green qPCR Kit (AG11701, Accurate Biotechnology). Furthermore, the relative expression level of mRNA was determined using the 2^−ΔΔCT^ approach, and U6 and TUBULIN were used as internal controls. All primers used in study were provided by Accurate Biotechnology (Additional file 1: Table S2).

### Cell transfection

HTR8/SVneo cells were transfected with miR-146a-5p mimic or inhibitor (100 nM) purchased from Beijing Syngentech (Beijing, China) following the guidelines for lipofectamine 2000 (Invitrogen, Carlsbad, CA, USA). Cells were grouped as NC mimic-, miR-146a-5p mimic-, NC inhibitor-, and miR-146a-5p inhibitor-treated groups. Furthermore, HTR8/SVneo cells pretreated with or without aPL were transfected with siTRAF6 or siNC (50 nM) purchased from Beijing Syngentech (Beijing, China) following the instructions for Lipofectamine 3000 (Invitrogen). The RNAi sequences utilized for cell transfection can been obtained in Additional file 1: Table S3.

### Animal model of antiphospholipid antibody syndrome pregnancy and therapeutic experiments

In vivo animal experiments were performed using C57BL/6 J mice (aged 8 weeks) purchased from Weitong Lihua (Beijing, China). All mice were housed in a breeding facility that was free from specific pathogens, following a 12-h cycle of light and darkness, and maintaining suitable temperature and humidity conditions. Female and male mice in the reproductive period mated at 18:00 on the previous day in a ratio of 2:1 or 3:1, and the vaginal plugs were checked at 8:00 the next day; if present, the mice were counted as E0.5 and then randomly distributed into three groups. At E0.5 and E7.5, the OAPS group was administered a tail vein injection of 100 μg/mouse anti-β2GPI antibody, while the exosome intervention group received a daily intravenous injection of 50 μg/mouse hucMSC-exos from E4.5 to E10.5 to ensure their sustained presence and efficacy in vivo. The sham group was administered sterile saline intravenously, with the quantity of injection being comparable to that of the anti-β2GPI antibody and hucMSC-exos. At E14.5, the mice were euthanized by dislocation of cervical vertebra following the American Veterinary Medical Association (AVMA) Guidelines for the Euthanasia of Animals (2020) and the uteri were dissected. The weights of embryos and placentas were measured, and the FRF (fetal resorption frequency) was calculated as the percentage of fetal resorptions out of the total number of all fetuses. Lastly, placental specimens were separated and stored at −80 °C or fixed in formaldehyde.

### Western blot

All proteins extracted from the exosomes, cells, and placentas were separated using sodium dodecyl sulfate–polyacrylamide gel electrophoresis (Epizyme, Shanghai, China) with corresponding proportions, following the principle of equal mass and volum. Next, the polyvinylidene fluoride membranes (Millipore, MA, USA) containing proteins were sealed in a protein-free fast-blocking solution at RT for 20 min and incubated with the primary antibodies overnight. The following day, after incubated with the corresponding secondary antibodies, the protein bands were photographed using an Amersham Imager 600 system (GE, Boston, MA, USA), and gray levels of the protein bands were determined and quantified using ImageJ software.

The information of antibodies: CD81 (ab109201, Abcam), TSG101 (ab125011, Abcam), GM130 (ab52649, Abcam), OCT4 (ab181557, Abcam), CD63 (ab134045, Abcam), BAX (ab128733, Abcam), BCL2 (ab182858, Abcam), Cleaved-CASP3 (#9664S, CST), IL-1β (ab254360, Abcam), IL-18 (ab191860, Abcam; ab207324, Abcam), TRAF6 (ab33915, Abcam), NF-κB p65 (ab32536, Abcam), and ACTIN (#3700S, CST).

### Hematoxylin and eosin and immunohistochemistry staining

After fixed and dehydrated successively, the paraffin-embedded placental tissue blocks were sliced thinly (4 μm) and subjected to H&E staining for histological examination.

For immunohistochemistry staining, the slices underwent dewaxing and hydration in ethanol solutions of varying concentrations (100%, 85%, and 75%) for a duration of 5 min each. Afterwards, the slides were placed in EDTA antigen retrieval solution (pH 8.0) to repair the antigens, and then submerged in 3% hydrogen peroxide to block endogenous peroxidase. Following incubation in a 5% solution of bovine serum albumin (BSA) for 30 min, the sections were incubated with the primary antibodies overnight at 4 °C. Subsequently, a secondary antibody and streptavidin-HRP (BioCare Medical) were applied, and the reaction was visualized using DAB peroxidase substrate reagent. Finally, the sections were stained with hematoxylin and imaged using a NIKON digital sight DS-FI2 (Nikon, Japan). MVD was calculated and observed under a light microscope for each section. The count units for blood vessels were determined by counting cells or cell clusters that expressed CD31. Using a low magnification (40 ×), we selected three regions with the highest MVD; then employing a high magnification (200 ×), we tailed the quantity of CD31 positive cells or cell clusters in five visual fields and calculated the average as the MVD for the specimen. Relative average density was quantified using ImageJ software.

The antibodies informations: CD31 (ab28364, 1:50, Abcam), TRAF6 (ab137452, 1:100, Abcam), and NF-κB p65 (ab32536, 1:1000, Abcam).

### Immunofluorescence staining

HTR8/SVneo cells under different interventions were successively fixed, permeabilized and blocked. The cells were then incubated with primary antibodies for a minimum of 18 h. The following day, cells were incubated with a secondary antibody in the dark. Immediately after the cytoskeleton and cell nucleus were stained with α-Smooth Muscle Actin Rabbit Alexa Fluor 555 conjugated (#60839, CST) and DAPI (Boster) respectively, the fluorescence signal was photographed using a Zeiss Cell Discoverer 7 with ZEN 3.1 professional analysis software.

Immunofluorescence staining was performed to assess the protein levels of the mouse placental tissue. The fluorescent graphics were collected using a NIKON digital sight DS-FI2 (Nikon, Japan). The relative fluorescence intensity was quantified using ImageJ software.

The primary antibodies used for immunofluorescence: Ki67 (ab15580, 0.5 µg/mL, Abcam), CD31 (ab28364, 1:50, Abcam), TRAF6 (ab137452, 1:100, Abcam), NF-κB p65 (ab32536, 1:1000, Abcam), and Alexa Fluor 488/594-conjugated goat anti-rabbit IgG (1:100, Bioss, Woburn, MA, USA).

### Fluorescence in situ hybridization

The placental tissue slices embedded in paraffin were heated at 65 °C for 2 h, dewaxed in xylene, and rehydrated in a range of graded ethanol. After incubating in proteinase K working solution (20 µg/mL) at 37 ℃ for permeabilization, the sections were hybridized in the hybridization buffer with the anti-sense probe against has-miR-146a-5p labeled with Cyanine 5 (500 nM), and the signal was amplified by incubating overnight at 40 ℃. Following a 10 min washing with sodium citrate buffers of 2 × concertration, 1 × concertration and 0.5 × concertration, the slices were then subjected to a 30 min incubated in BSA at RT. Afterward, the slices were observed under a NIKON digital sight DS-FI2 (Nikon, Japan), and images were collected.

### Statistical analysis

GraphPad Prism software 8.0 was used for statistical analyses. A two-tailed unpaired Student’s t-test was performed for comparison between two groups. One-way ANOVA was used to compare among three or more groups. The data are expressed as the means ± SD and P < 0.05 was considered statistically significant (*P < 0.05, **P < 0.01, ***P < 0.001, ****P < 0.0001).

### Supplementary Information

**Additional file 1: Table S1**. Demographic informations of samples enrolled in this study.** Table S2**. Primers used in this study for qRT-PCR.** Table S3**. The sequences of RNAi used in this study for cell transfection.** Figure S1**. Flowchart of extracting exosomes by ultracentrifugation. The culture supernatant was collected in 50 ml centrifuge tube and centrifuged at 300 × g for 10min, 2000 × g for 30 min, and 12,000 × g for 45 min to separate cell debris and other macromolecules. The resultant supernatant fluid was passed through a 0.22 μm sterilefilter (Steritop; Millipore) and then ultracentrifuged at 120,000 × g for 70 min twice(Beckman Coulter). Finally, the supernatant was discarded and the precipitation was the hucMSC-exos.** Figure S2**. Immunofluorescence analyzed the nuclear translocation of NF-κB p65 in the human placenta. (A and B) Representative low- and high-magnification images of NF-κB p65 IF staining in the placenta of normal health pregnant women and patients with OAPS. (NF-κB, red; DAPI nuclear stain, blue), quantified by fluorescence intensity (n=5). Scale bars, 100 μm and 20 μm. *p < 0.05, **p < 0.01, ***p < 0.001, ****p < 0.0001.** Figure S3**. Technical route of preparation of anti-β2GPI antibody. We have collaborated with Affinity Biosciences to develop the anti-β2GPI antibody. First, the polypeptide sequence (GRTCPKPDDLPF) was determined using AbDesigner software. After coupling with immune antigens, the polypeptides were used to immunize the Bal b/c mice. Then, the serum of mice that underwent four rounds of booster immunization was collected to screen out successfully immunized mice. The spleen cells of immunized mice and the SP20 cells were mixed and the hybridoma cells were screened by ELISA to establish a monoclonal cell bank. The hybridoma cells were inoculated into the abdominal cavity of mice and ascites were collected to identify and purify the monoclonal antibodies.

## Data Availability

The datasets generated and analyzed during present study can be obtained from corresponding authors according to reasonable requirements.

## Abbreviations

HucMSC-exos: Human umbilical cord mesenchymal stem cells
TRAF6: Tumor necrosis factor receptor-associated factor 6
IL-1β: Interleukin-1β
IL-18: Interleukin-18
aPLs: Antiphospholipid antibodies
OAPS: Obstetric antiphospholipid syndrome
β2GPI: β2-Glycoprotein I
TEM: Transmission electron microscop
NTA: Nanoparticle tracking analysis
FISH: Fluorescence in situ hybridization
FRF: Fetal resorption frequency
DAPI: 4',6-Diamidino-2-phenyl indole
MVD: Microvessel density
