# Supplementary material for: Exosomal miR-146a-5p derived from human umbilical cord mesenchymal stem cells can alleviate antiphospholipid antibody-induced trophoblast injury and placental dysfunction by regulating the TRAF6/NF-κB axis
Source: J Nanobiotechnology. 2023 Nov 13;21:419. doi: 10.1186/s12951-023-02179-5 (PMC10641965; doi:10.1186/s12951-023-02179-5)
Supplement: Supplementary file 1 — Additional file 1: Table S1. Demographic informations of samples enrolled in this study. Table S2. Primers used in this study for qRT-PCR. Table S3. The sequences of RNAi used in this study for cell transfection. Figure S1. Flowchart of extracting exosomes by ultracentrifugation. The culture supernatant was collected in 50 ml centrifuge tube and centrifuged at 300 × g for 10min, 2000 × g for 30 min, and 12,000 × g for 45 min to separate cell debris and other macromolecules. The resultant supernatant fluid was passed through a 0.22 μm sterilefilter (Steritop; Millipore) and then ultracentrifuged at 120,000 × g for 70 min twice(Beckman Coulter). Finally, the supernatant was discarded and the precipitation was the hucMSC-exos. Figure S2. Immunofluorescence analyzed the nuclear translocation of NF-κB p65 in the human placenta. (A and B) Representative low- and high-magnification images of NF-κB p65 IF staining in the placenta of normal health pregnant women and patients with OAPS. (NF-κB, red; DAPI nuclear stain, blue), quantified by fluorescence intensity (n=5). Scale bars, 100 μm and 20 μm. *p < 0.05, **p < 0.01, ***p < 0.001, ****p < 0.0001. Figure S3. Technical route of preparation of anti-β2GPI antibody. We have collaborated with Affinity Biosciences to develop the anti-β2GPI antibody. First, the polypeptide sequence (GRTCPKPDDLPF) was determined using AbDesigner software. After coupling with immune antigens, the polypeptides were used to immunize the Bal b/c mice. Then, the serum of mice that underwent four rounds of booster immunization was collected to screen out successfully immunized mice. The spleen cells of immunized mice and the SP20 cells were mixed and the hybridoma cells were screened by ELISA to establish a monoclonal cell bank. The hybridoma cells were inoculated into the abdominal cavity of mice and ascites were collected to identify and purify the monoclonal antibodies. [file 12951_2023_2179_MOESM1_ESM.pdf]

Table S1 Demographic informations of samples enrolled in this study

Variables	NH	OAPS
Number (n)	n=25	n=25
Age (years)	27.32±2.85	28.76±3.02
Weight (kg)	59.54±3.77	59.80±4.94
Height (cm)	163.58±3.38	163.91±4.44
Gestational weeks (w)	12.43±0.64	12.39±0.90
Clinical manifestations：		
Recurrent spontaneous abortions	0 (0 %)	21 (84 %)
Unexplained stillbirth	0 (0 %)	5 (20 %)
FGR	0 (0 %)	2 (8 %)
Laboratory indicators：positive (% patients)		
Lupus anticoagulant	0 (0 %)	5 (20 %)
anti-β2GPI IgG	0 (0 %)	25 (100 %)
anti-β2GPI IgM	0 (0 %)	7 (25 %)
aCL IgG	0 (0 %)	12 (48 %)
aCL IgM	0 (0 %)	6 (24 %)

Abbreviations: NH: normal health; OAPS: obstetric antiphospholipid antibody syndrome; FGR: foetal growth restriction; β2GPI: β2-glycoprotein I; aCL: anti-cardiolipin antibodies.

Values are mean ± S.D.

Table S2 Primers used in this study for qRT-PCR

Gene		5'- Primer -3'
hsa-miR-21-5p	F	TAGCTTATCAGACTGATGTTGA
hsa-miR-27b-3p	F	GGTTCACAGTGGCTAAGTTCT
hsa-miR-146a-5p	F	GGTCGCATCGACGATCAGCCA
hsa-miR-125b-5p	F	TCCCTGAGACCCTAACTTGTGA
hsa-miR-143-3p	F	GCGCTGAGATGAAGCACTG
hsa-miR-100-5p	F	CCCGTAGATCCGAACTTGTG
hsa-miR-221-3p	F	CTACATTGTCTGCTGGGTTTC
hsa-miR-320-3p	F	AAAAGCTGGGTTGAGAGGGCGA
hsa-miR-127-3p	F	CCGAATCTGTGCTTGGCTAAA
hsa-miR-423-5p	F	AGGGGCAGAGAGCGAGACTTT
HumanTUBB3 (TUBULIN)	F	GGCCAAGGGTCACTACACG
	R	GCAGTCGCAGTTTTCACACTC
human TRAF6	F	ATGCGGCCATAGGTTCTGC
	R	TCCTCAAGATGTCTCAGTTCCAT
human IL-1β	F	ATGATGGCTTATTACAGTGGCAA
	R	GTCGGAGATTCGTAGCTGGA
human IL-18	F	TCTTCATTGACCAAGGAAATCGG
	R	TCCGGGGTGCATTATCTCTAC
human CASPASE 3	F	CATGGAAGCGAATCAATGGACT
	R	CTGTACCAGACCGAGATGTCA
human BAX	F	CCCGAGAGGTCTTTTTCCGAG
	R	CCAGCCCATGATGGTTCTGAT
human BCL2	F	GGTGGGGTCATGTGTGTGG
	R	CGGTTCAGGTACTCAGTCATCC

Table S3 The sequences of RNAi used in this study for cell transfection

	Sequences (5’-3’)
hsa-miR-146a-5p mimic	UGAGAACUGAAUUCCAUGGGUU
hsa-miR-146a-5p inhibitor	TAGCTTATCAGACTGATGTTGA
NC mimic	GCTCCTCTTAGGGGCCACT
NC inhibitor	GGTCGCATCGACGATCAGCCA
hTRAF6 si-1	GCCUAAUCAUUAUGAUCUATT
hTRAF6 si-2	CGGAAUUUCCAGGAAACUAUUTT
hTRAF6 si-3	UGGGAUUCUACACUGGCAAATT

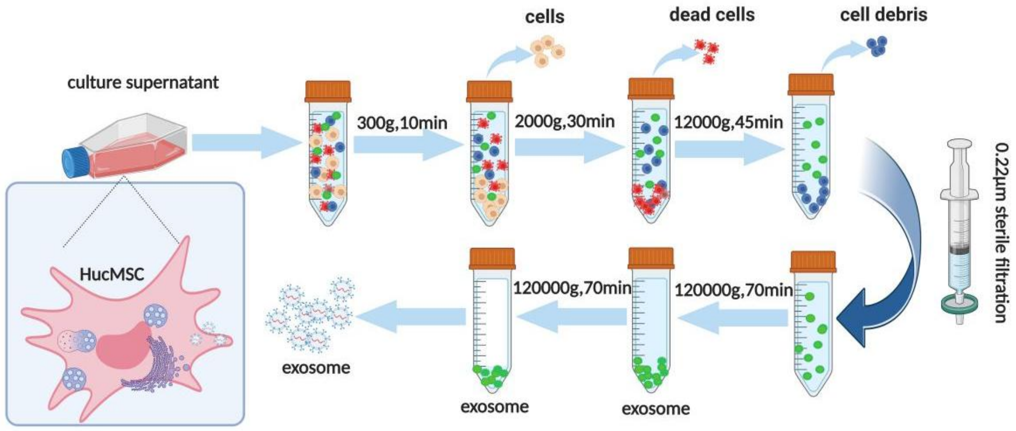

Figure S1 Flowchart of extracting exosomes by ultracentrifugation. The culture supernatant was collected in 50 ml centrifuge tube and centrifuged at 300 × g for 10 min, 2000 × g for 30 min, and 12,000 × g for 45 min to separate cell debris and other macromolecules. The resultant supernatant fluid was passed through a 0.22 μm sterile filter (Steritop; Millipore) and then ultracentrifuged at 120,000 × g for 70 min twice (Beckman Coulter). Finally, the supernatant was discarded and the precipitation was the hucMSC-exos.

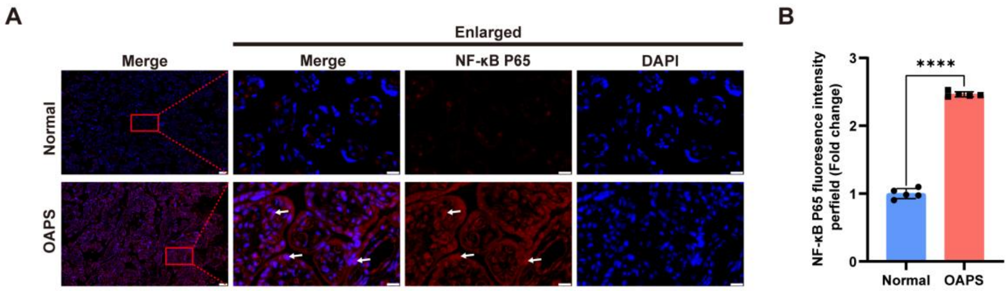

Figure S2 Immunofluorescence analyzed the nuclear translocation of NF-κB p65 in the human placenta. (**A** and **B**) Representative low- and high-magnification images of NF-κB p65 IF staining in the placenta of normal health pregnant women and patients with OAPS. (NF-κB, red; DAPI nuclear stain, blue), quantified by fluorescence intensity (n=5). Scale bars, 100 μm and 20 μm. *$p < 0.05$, **$p < 0.01$, ***$p < 0.001$, ****$p < 0.0001$.

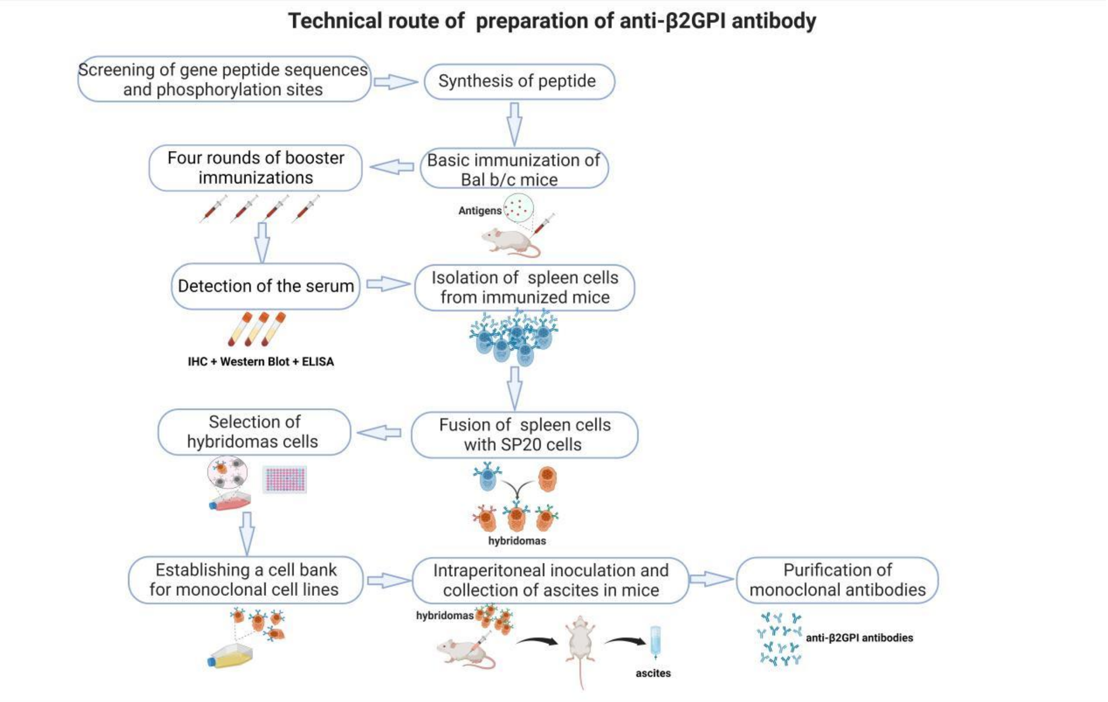

Figure S3 Technical route of preparation of anti-β2GPI antibody. We have collaborated with Affinity Biosciences to develop the anti-β 2GPI antibody. First, the polypeptide sequence (GRTCPKPDDLPF) was determined using AbDesigner software. After coupling with immune antigens, the polypeptides were used to immunize the Bal b/c mice. Then, the serum of mice that underwent four rounds of booster immunization was collected to screen out successfully immunized mice. The spleen cells of immunized mice and the SP20 cells were mixed and the hybridoma cells were screened by ELISA to establish a monoclonal cell bank. The hybridoma cells were inoculated into the abdominal cavity of mice and ascites were collected to identify and purify the monoclonal antibodies.
